# Improving amphibian genomic resources: a multitissue reference transcriptome of an iconic invader

**DOI:** 10.1093/gigascience/gix114

**Published:** 2017-11-27

**Authors:** Mark F Richardson, Fernando Sequeira, Daniel Selechnik, Miguel Carneiro, Marcelo Vallinoto, Jack G Reid, Andrea J West, Michael R Crossland, Richard Shine, Lee A Rollins

**Affiliations:** 1 Deakin University, Bioinformatics Core Research Group, 75 Pigdons Road, Locked Bag 20000, Geelong, VIC 3220, Australia; 2 Deakin University, School of Life and Environmental Sciences, Centre for Integrative Ecology (Waurn Ponds Campus), 75 Pigdons Road, Locked Bag 20000, Geelong, VIC 3220, Australia; 3 CIBIO-InBIO, Centro de Investigação em Biodiversidade e Recursos Genéticos, Campus Agrário de Vairão, Universidade do Porto, 4485-661, Vairão, Portugal; 4 Departamento de Biologia, Faculdade de Ciências, Universidade do Porto, Rua do Campo Alegre s/n., 4169-007 Porto, Portugal; 5 Laboratório de Evolução (LEVO), Instituto de Estudos Costeiros (IECOS), Universidade Federal do Pará, Campus de Bragança, Pará, Brasil; 6 School of Life and Environmental Sciences, The University of Sydney, NSW 2006, Australia

**Keywords:** *de novo* assembly, *Bufo marinus*, cane toad, *Rhinella marina*, invasive species, RNA-Seq, transcriptome, anuran, amphibian

## Abstract

**Background:**

Cane toads (*Rhinella marina*) are an iconic invasive species introduced to 4 continents and well utilized for studies of rapid evolution in introduced environments. Despite the long introduction history of this species, its profound ecological impacts, and its utility for demonstrating evolutionary principles, genetic information is sparse. Here we produce a *de novo* transcriptome spanning multiple tissues and life stages to enable investigation of the genetic basis of previously identified rapid phenotypic change over the introduced range.

**Findings:**

Using approximately 1.9 billion reads from developing tadpoles and 6 adult tissue-specific cDNA libraries, as well as a transcriptome assembly pipeline encompassing 100 separate *de novo* assemblies, we constructed 62 202 transcripts, of which we functionally annotated ∼50%. Our transcriptome assembly exhibits 90% full-length completeness of the Benchmarking Universal Single-Copy Orthologs data set. Robust assembly metrics and comparisons with several available anuran transcriptomes and genomes indicate that our cane toad assembly is one of the most complete anuran genomic resources available.

**Conclusions:**

This comprehensive anuran transcriptome will provide a valuable resource for investigation of genes under selection during invasion in cane toads, but will also greatly expand our general knowledge of anuran genomes, which are underrepresented in the literature. The data set is publically available in NCBI and *Giga*DB to serve as a resource for other researchers.

## Data Description

### Background

It is well established that genome size across taxa is related to repetitive DNA content [1]. Highly repetitive genomes present significant challenges to genome assembly [2], which likely accounts for the scarcity of large genome sequences currently available. Anuran genome size is highly variable (C-values of 0.95–13.02) [3], and to date, genome sequences of only 3 anurans have been published: *Xenopus tropicalis* [4], *X. laevis* [5], and *Nanorana parkeri* [6]. Large genomes typify many Bufonids, including the cane toad (*Rhinella marina*; average reported C-value = 4.79) [3], and none have been sequenced to date. Transcriptome sequencing provides a tenable alternative to genome sequencing in anurans because the large, repetitive, noncoding regions typical of their large genomes are not sequenced [7].

**Figure 1:**
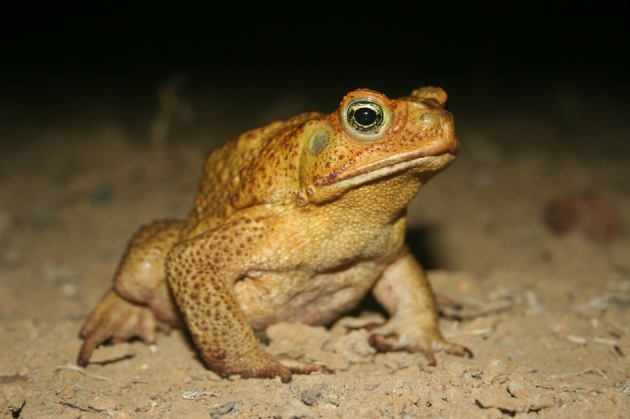
The cane toad, *Rhinella marina*. NCBI *Taxonomy ID*: 8386. Photographer credit: Matt Greenlees. Source: Matt Greenlees.

Cane toads (NCBI Taxonomy ID: 8386) (Fig. 1) are an excellent model for the study of invasion. Because they were intentionally and repeatedly introduced to novel environments as a biocontrol agent, their introduction history is well documented [8]. A wealth of evolutionary and ecological knowledge about cane toads currently exists, documenting phenotypic evidence of rapid evolution in introduced environments, but genomic data are scarce [9]. Providing access to well-developed genomic resources for the cane toad will enable the investigation of the genetic basis of traits underlying invasion ability in this species, which will in turn significantly advance our understanding of invasion genetics for all species. Here we present a *de novo* transcriptome assembly covering multiple *R. marina* tissues and life stages, representing one of most complete anuran genomic resources reported to date.

### Samples

Cane toad samples and tissues (7 in total) used in this study were obtained from several sources within the invasive (Australian) and native (Brazilian) range. Several different methods were used to prepare and sequence samples; for simplicity, we describe the samples and data sources used based on the tissue types sequenced (Table 1).

**Table 1:** Cane toad samples used to generate the *de novo* reference transcriptome

Tissue	Origin	Platform	Sample ID (library size)	Sampling location	Sex	SRA
Brain	Australia	HiSeq 2500	B19 (23.9 M)	Durack	F	SRR5446736
		(2 × 125 bp)	B20 (27.7 M)	Durack	F	SRR5446735
			B31 (24.8 M)	Gordonvale	F	SRR5446734
			B32 (22.3 M)	Gordonvale	F	SRR5446733
Spleen	Australia	HiSeq 2500	S1 (23.8 M)	Gordonvale	F	SRR5446732
		(2 × 125 bp)	S2 (25.0 M)	Gordonvale	F	SRR5446732
			S18 (24.7 M)	Durack	F	SRR5446732
			S19 (23.6 M)	Durack	F	SRR5446732
Muscle	Australia	HiSeq 2000	RM0021M (93.8 M)	El Questro	F	SRR1910534
		(2 × 100 bp)				SRR1910535
			RM0094M (88.2 M)	Purnululu	F	SRR1910543
			RM0108M (97.6 M)	Innisfail	F	SRR1910545
			RM0169M (80.0 M)	Rossville	F	SRR1910549
Tadpole	Australia	HiSeq 2500	T1 (26.4 M)	Oombulgurri	Both	SRR5446728
		(2 × 125 bp)	T4 (24.5 M)	Oombulgurri	Both	SRR5446727
			T7 (23.2 M)	Innisfail	Both	SRR5446726
			T10 (25.7 M)	Innisfail	Both	SRR5446725
Liver	Brazil	HiSeq 2000	RMTP (536.8 M)	Macapá	NA	SRR1514601
		(2 × 75bp)				
Ovary	Brazil	HiSeq 1500	AR19 (434.1 M)	Macapá	F	SRR5446724
		(2 × 125 bp)				
Testes	Brazil	HiSeq 1500	AR05 (410.5 M)	Macapá	M	SRR5446723
		(2×125 bp)				

Library size is given as raw sequenced reads in millions (M), sex denoted as female (F) and male (M). Both: sample contains mixed individuals of both sexes; NA: information unknown.

*Brain and spleen*

Four adult female toads were collected across 2 sites in Australia, 2 from Durack (15.9419°S, 127.2202°E), Western Australia, and 2 from Gordonvale (17.0972°S, 145.7792°E), Queensland, in May 2015. Toads were euthanized using lethal injection of 150 mg/kg sodium pentobarbital, and the whole brain and spleen were harvested and immediately stored in RNAlater (Qiagen, USA), kept at 4°C, then transferred to –80°C for storage until RNA extraction.

*Tadpoles*

We conducted a tadpole rearing experiment in March 2015. Four adult toads (2 males and 2 females) were collected from both Oombulgurri (15.1818°S, 127.8413°E), Western Australia, and Innisfail (17.4963°S, 146.0465°E), Queensland, Australia. To obtain egg clutches, pairs of adult male and female toads per population (i.e., 2 separate male × female crosses) were subcutaneously injected with 0.25 mg/mL Leuproelin acetate (Lucrin Abbott Australasia, Kurnell, Australia) in amphibian Ringer's solution to stimulate spawning; males received 0.25 mL and females 0.75 mL. The pairs of male and female toads were left overnight in 750-L plastic enclosures that contained bore water to lay and fertilize egg clutches. Egg clutches were removed and placed in 17-L tanks containing continuously aerated bore water and monitored to ensure fertilization had occurred. Embryos were selected once they reached Gosner stage 16–17 [10]. Three replicates of 5 fertilized embryos were removed per clutch and placed in 1-L containers, each with 750 mL bore water, where they were raised until 10 days old; water was changed daily, and developing tadpoles were fed 12 mg of a commercial algae supplement (Hikari algae pellets, Kyorin, Himeji, Japan) after each water change. One tadpole from each of the 3 replicate tadpole tanks per clutch was euthanized (immersion in 2g/L Tricaine methanesulfonate) and immediately stored in RNAlater, kept at 4°C, then transferred to –80°C for storage until RNA extraction.

Total RNA was extracted from each of the brain, spleen, and tadpole samples using Qiagen RNeasy kits (Qiagen, USA), following the manufacturers protocol with an additional DNase digestion step. Extracted RNA was quantified using a Quibit RNA HS assay on a Qubit 3.0 Fluorometer (Life Technologies, USA). For the tadpole sequencing, total RNA from the 3 “replicate” tadpoles per clutch was pooled in equal quantities, resulting in 4 pooled samples. Two μg of total RNA per sample was sent to Macrogen (Macrogen Inc., Seoul, ROK), where mRNA libraries were constructed using the TruSeq mRNA v2 sample kit (Illumina Inc, San Diego, CA, USA), which included a 300-bp size selection step. Libraries were sequenced on 1 lane of Illumina HiSeq 2500 (Illumina Inc, San Diego, CA, USA), generating 295.6 million paired-end 2×125-bp reads. Raw reads are available in the NCBI Short Read Archive (SRA) under the Bioproject Accession PRJNA382870.

*Muscle*

We downloaded raw fastq files (NCBI Bioproject Accession: PRJNA277985; paired-end 2×100 bp; Illumina HiSeq-2000) for 4 adult female toads (RM0021M, RM0094M, RM0108M, and RM0169M) across 4 populations in Australia (El Questro, 16.007872S, 128.020494E; Purnululu National Park, 17.4334°S, 128.3018°E, both Western Australia; Innisfail, 17.4963°S, 146.0465°E; Rossville, 15.7054°S, 145.2229°E, both Queensland) previously used to build a *de novo* muscle (*triceps femoris*) transcriptome [9].

*Ovary and testes*

Two adult toads (1 male and 1 female) were collected from Macapá (0.0432°S, 51.1241°W), Amapá, Brazil, in December 2015. Toads were euthanized as described above, and ovary and testes were excised and immediately stored in RNAlater, then kept at 4°C for storage until RNA extraction. Total RNA was extracted using Qiagen RNeasy kits, following the manufacturer’s protocol with an additional DNase digestion step. Extracted RNA was quantified using a Qubit RNA BR assay, and RNA integrity was assessed using a Tapestation 2200 (Aligent Tech., Santa Clara, CA, USA) with an RNA screen. One μg of total RNA per sample was used to construct mRNA libraries using the TruSeq mRNA v2 sample kit, which included a 130–350-bp size selection step. Both libraries were run on a HiSeq 1500 using Illumina V4 PE chemistry across 2 lanes (1 lane for each sample), generating 844.6 million paired-end 2×125-bp reads. Raw reads are available in the NCBI SRA under the Bioproject Accession PRJNA382870.

*Liver*

We downloaded raw fastq files (NCBI Bioproject Accession: PRJNA255079; paired-end 2×75 bp; Illumina HiSeq-2000) from a pool of 5 adult toads from Macapá, Amapá, Brazil, previously used to build a *de novo* liver transcriptome [11].

### Data preprocessing and multiple *de novo* transcriptome assemblies

Raw reads from each sample were first processed with Trimmomatic v0.33 [12], using the following parameters: ILLUMINACLIP: TruSeq3-PE.fa:2:30:10:4 HEADCROP:13 AVGQUAL:30 MINLEN:36, to (i) remove adaptor sequences, (ii) trim the first 13 bp of a read, (iii) discard reads with an average quality <Phred 30, and (iv) remove reads <36 bp after processing. We then concatenated reads into 2 input data sets, 1 containing all samples from Australia and 1 containing those from Brazil. To reduce the computational load of assembly, we used the *in silico* normalization approach implemented in Trinity v2.1.1 (Trinity, RRID:SCR_013048) [13], with *–normalize_max_read_cov = 50*, on both of the input data sets. The normalized Australia and Brazil data sets contained ∼42.2 million and ∼82.2 million reads, respectively. Multiple independent *de novo* transcriptome assemblies were conducted for each of the input data sets, resulting in 100 separate assemblies (Table 2). In brief, we used 3 assemblers: Trinity, with default parameters and *–min_contig_length = 300*; SOAPdenovo-Trans v1.03 (SOAPdenovo-Trans, RRID:SCR_013268) [14], with 13 different k-mers (apart from the Brazil input set, which had 12) for each combination of *EdgeCovCutoff = 2, mergeLevel = 1, EdgeCovCutoff = 3, mergeLevel = 2*; the parameters -f, -F, and *minContigLen = 200* were the same for all assemblies; velvet v1.2.09/oases v0.2.08 (Velvet, RRID:SCR_010755/Oases, RRID:SCR_011896) [15, 16], with 12 different k-mers for each combination of -*cov_cutoff = 3, -min_pair_count = 4*, and -*cov_cutoff = 5, -min_pair_count = 6*, where *-ins_length = 300* and *-min_trans_lgth = 200*, were consistent across assemblies. The individual assemblies were then compiled into an “over-assembly” of ∼42 million transcripts. To reduce redundancy in the “over-assembly,” we used the tr2aacds pipeline from the Evidential Gene package [17], which selects the “optimal” set of transcripts based on their coding potential. This reduced the redundant “over-assembly” to the final assembly of 62 202 transcripts. Of these, 50% (31 040) are commonly expressed among the 7 different tissues used in the assembly, while a total of 6.64% exhibit tissue-specific expression (Fig. S1: Additional file 1) [32]. We then used TransDecoder v3.0.0 to predict protein coding sequences (CDS) with a minimum CDS of 100 bp. Transvestigator [18] was used to prepare the final assembly for submission to NCBI’s Transcriptome Shotgun Assembly (TSA) database—accessible through the PRJNA383966 accession. Results from the assembly pipeline are described in Table 3. As the “dropset”—those transcripts not kept in the “optimal” tr2aacds output—may contain other biologically relevant transcripts, such as noncoding RNAs and active transposable elements, we also provide these transcripts in the associated *Giga*DB repository [32].

**Table 2:** *De novo* assembler parameters used to produce the “over-assembly”

Assembler	*k*-mers	Parameter combinations	No. of assemblies
Trinity	25	Default	Aus 1, Brazil 1
SOAPdenovo-Trans	21, 25, 29, 33, 37, 41, 45, 49, 59, 69, 79, 89, 99 (No. 99 for the Brazil input set)	*EdgeCovCutoff = 2* and *mergeLevel = 1; EdgeCovCutoff = 3* and *mergeLevel = 2*	Aus 13, Brazil 12; Aus 13, Brazil 12
Velvet/Oases	21, 25, 29, 33, 37, 41, 45, 49, 59, 69, 79, 89	*cov_cutoff = 3* and *min_pair_count = 4; cov_cutoff = 5* and *min_pair_count = 6*	Aus 12, Brazil 12; Aus 12, Brazil 12
			Total: 100

**Table 3:** Summary of transcriptome assembly and annotation statistics compared with previous cane toad transcriptomes

	This study	Muscle^a^	Liver^b^
Assembly			
Filtered read pairs	945 348 780	99 462 214	265 684 605
*In silico* normalized reads	129 051 008	18 713 526	-
Assembly size, bp	83 724 193	60 388 685	80 251 892
Number of transcripts	62 202	57 580	131 020
N50	2377	1871	916
Average length, bp	1346	1049	613
Minimum length, bp	297	201	201
Maximum length, bp	99 438	40 546	17 369
Median length, bp	698	577	331
GC, %	46.05	45.06	44.32
Transcripts with CDS	62 202	19 751	–
Annotation			
Transcripts with BLASTx hit	31 103	21 533	–
Transcripts with BLASTp hit	28 560	16 754	–
Transcripts with GO terms	28 399	19 500	–

^a^Rollins, Richardson, and Shine [9].

^b^Arthofer et al. [11].

### Annotation

We conducted functional annotation based on our predicted protein sequences utilizing the automated Trinotate pipeline. Transcripts were first annotated based on sequence homology, where assembled nucleotides and translated CDS sequences were used in BLASTx (BLASTX, RRID:SCR_001653) and BLASTp (BLASTP, RRID:SCR_001010) searches, against the UniProt/Swiss-Prot database (downloaded Feb. 2017) using a standalone version of BLAST v2.2.26+ [19], with an e-value cutoff of 1×10^−5^. Pfam (Pfam, RRID:SCR_004726) [20] functional domains (downloaded Feb. 2017) were identified in protein coding sequences using hmmscan [21]; signal peptides and transmembrane domains were assigned using hidden Markov model prediction implemented in SignalP v4.1 [22] and TMHMM v2.0c [23], respectively. Finally, transcripts were compared with curated annotations in the eggNOG (eggNOG, RRID:SCR_002456) [24] and Gene Ontology (GO, RRID:SCR_002811) [25] databases. A summary of annotation metrics is provided in Table 3. The combined Trinotate functional annotations to the TSA assembly are available in the associated *Giga*DB [26].

### Quality and completeness of the cane toad transcriptome

To evaluate our new multitissue transcriptome assembly, we used 3 comparative approaches to assess relative quality and completeness. First, we compared core assembly statistics of the new assembly to our 2 previous cane toad single-tissue transcriptomes derived from muscle and liver tissue (Table 3). The inclusion of data from multiple tissues (encompassing 9.5- and 3.5-fold increases in read input compared with the muscle and liver transcriptomes, respectively) resulted in increases of all assembly metrics, apart from the number of assembled transcripts, which fell compared with the liver transcriptome (Table 3). Notably, mean transcript length increased from 613 (liver) to 1346 bp, and transcript n50 increased from 916 (liver) to 2377 bp. The total assembled bases were similar between the multitissue transcriptome and that assembled from liver, yet higher (∼20 million bp) than that produced from muscle tissue. Additionally, our assembly exhibits comparable lengths of mRNAs and CDS to those from *X. tropicalis* (gene build v9.0, downloaded Xenbase.org, Aug. 2017) (Fig. S2: Additional file 1), albeit with a greater frequency of shorter features. This is not unexpected given that we are comparing a *de novo* transcriptome assembly to gene models from a genome assembly. Also, our assembly shows good coverage of the lengths of CDS features compared with *X. tropicalis* (Fig. S3: Additional file 1), given both species are substantially divergent. Importantly, the new *R. marina* multitissue assembly increases the coverage of transcripts containing protein coding sequences with associated BLAST matches and Gene Ontology annotations compared with the previously available *R. marina* assemblies.

Second, we evaluated the new assembly using the Benchmarking Universal Single-Copy Orthologs (BUSCO) vertebrate gene set (BUSCO, RRID:SCR_015008) [27], which uses 3023 near-universal orthologs (hereafter BUSCOs) to evaluate the relative completeness of assemblies and compares the results with the previous *R. marina* assemblies and those of several available amphibian transcriptomes and genomes (Table 4). We used BUSCO v1.2 [27] with the default e-value cutoff of 0.01 and *–mode = Trans* for all the transcriptomes compared and *–mode = OGS* for the genome comparisons (using the *N. parkeri* v2.0, *X. tropicalis* v9.0 and *X. laevis* v9.1_1.8.3.2 gene builds downloaded from Xenbase.org, Aug. 2017). Our multitissue assembly had a much higher percentage of complete BUSCOs (90%), apart from the 2 *Xenopus* genomes, which exhibited comparable results (*X. tropicalis*, 91% and *X. laevis*, 97%). Additionally, our multitissue transcriptome has low BUSCO missingness, intermediate duplication of complete BUSCOs, and the second lowest level of fragmented BUSCOs. In contrast to the previous *R. marina* transcriptomes specifically, the new assembly has less fragmented and 20%–30% more complete BUSCO genes—suggesting the presence of more full-length transcripts. Overall, the comparison of BUSCO results revealed our assembly to be one of the most complete references available for anurans.

**Table 4:** BUSCO analysis of transcriptome completeness

	Complete	Complete and duplicated	Fragmented	Missing
	BUSCOs, %	BUSCOs, %	BUSCOs, %	BUSCOs, %
*R. marina* transcriptomes				
This study	90	4.7	1.7	7.8
Muscle^a^	60	4.6	5.7	33
Liver^b^	69	0.6	4.1	26
Select anuran transcriptomes				
*Bufotes viridis*^c^	26	0.3	15	57
*Rana catesbeiana*^d^	79	42	2.8	17
*Pelohylax nigromaculatus*^e^	50	0.4	7.8	41
*Microhyla fissipes*^f^	73	1.2	4.7	21
Select anuran genomes				
*Xenopus laevis*^g^	97	51	1.4	1.4
*Xenopus tropicalis*^h^	91	4.1	3.7	4.9
*Nanorana parkeri*^i^	76	2.8	9.0	14

“Complete BUSCOs” refers to those with a full-length match in the assembly. “Complete and duplicated” refers to those BUSCOs that are complete within an assembly but have multiple matches present. “Fragmented” are those BUSCOs that only have a partial match in the assembly, and “Missing” refers to those BUSCOs that do not have a corresponding match in the assembly.

^a^Rollins, Richardson, and Shine [9].

^b^Arthofer et al. [11].

^c^Gerhchen et al. [7].

^d^Birol et al. [28].

^e^Huang et al. [29].

^f^Zhao et al. [30].

^g^Session et al. [5].

^h^Hellsten et al. [4].

^i^Sun et al. [6].

Third, we compared the multitissue transcriptome with the previous cane toad transcriptomes and the 3 currently available Anuran genomes through both standard and reciprocal best-hit BLAST approaches. The standard approach revealed that 40 741 (65.5%) and 31 189 (50.1%) of our new assembly had significant matches (e-value < 10^−3^) to the liver and muscle transcriptomes, respectively. The reciprocal best-hit approach reduced the number of significant matches to both the liver (23 943; 38.5%) and muscle (15 892; 25.5%) transcriptomes, which may in part be due to transcripts mapping to multiple isoforms in the different assemblies. This, together with the high number of protein-coding transcripts in the multitissue assembly, indicates that the new assembly still contains some redundancy and that we have assembled multiple transcript variants for some genes. Standard BLAST comparisons of our assembly with the *X. tropicalis, X. laevis*, and *N. parkeri* proteins exhibited 40 275 (64.7%), 40 218 (64.7%), and 40 244 (64.7%) significant matches, respectively; 37 064 of our assembled transcripts with significant matches are common to all 3 species (Fig. S4: Additional file 1). Of our 31 103 assembled transcripts with annotations, 97.8% (30 423) have significant matches to *X. tropicalis*, while 97.9% (30 465) have matches to *X. laevis* and 98.3% (30 574) to *N. parkeri*; 96.3% (29 967) are common to all 3 species (Fig. S5: Additional file 1). The high percentage of annotated transcripts with matches to *X. tropicalis, X. laevis*, and *N. parkeri* provides further evidence that our assembly pipeline produced transcripts with strong anuran homology.

### Identification of anuran orthologues

Current efforts to identify amphibian-specific genes have been hampered by a lack of high-quality full-length genes for numerous amphibian species [31]. So far, the identification of amphibian-specific genes has not been possible as orthologous counterparts have only been identified between the 2 *Xenopus* genomes. We used OrthoFinder v1.1.10 [32] with default parameters to identify orthologues between our newly assembled *R. marina* CDS-containing transcripts and the proteomes from *N. parkeri, X. tropicalis*, and *X. laevis* (using the same gene build versions as in the BUSCO analysis). OrthoFinder identifies “orthogroups” (a group of genes descended from a single gene in the last common ancestor of a group of species) [32] and then orthologues between each pair of species in the comparison. Because OrthoFinder classifies genes with multiple orthologues (i.e., many to many relationships) in “orthogroups,” it may reduce the impact that multiple isoforms of the same gene have in such analyses. We assigned 60.2% (94 516) of all the genes examined to 18 776 “orthogroups” (see Additional file 2), of which 50% of all genes were found in “orthogroups” with 4 or more genes. Additionally, we identified 12 674 “orthogroups” that contained genes from all 4 species, and 4586 of these consisted entirely of single-copy genes. The data set presented here may be useful for further research into identifying amphibian-specific genes, so we have included this analysis in its entirety in the associated *Giga*DB repository [25].

### Conclusions

This comprehensive anuran transcriptome will not only serve as a valuable reference for investigation of genes under selection during invasion in cane toads, but will also expand our general knowledge of anuran genomes. Additionally, we have identified numerous orthologous transcripts to *X. tropicalis, X. laevis*, and *N. parkeri* proteins, which may aid the identification of amphibian-specific genes—an important objective of AmphiBase [31].

### Availability of supporting data

The data sets supporting the results presented here are available in the associated *Giga*DB repository [25]. All raw sequencing data used in this study are available in the SRA and associated with the following BioProject accessions: PRJNA277985, PRJNA255079, PRJNA382870, and PRJNA383966. The final transcriptome assembly has been deposited at DDBJ/EMBL/GenBank under the accession GFMT00000000. The version described in this paper is the first version, GFMT01000000.

### Additional file

Additional file 1: Figure S1: Schematic diagram showing the percentage (%) of expressed transcripts among the 7 different tissues used in the assembly. For brevity, we only show those common to all tissues (centre of elements) and those uniquely expressed in each separate tissue. Transcript expression was quantified using Salmon v0.8.0 [1] using *–l IU* and default parameters. Additionally, detailed comparative analysis of expression among all tissue combinations is provided in the associated *Giga*DB repository [2].

Additional file 1: Figure S2: Histogram of the lengths of *R. marina* assembled mRNAs and CDS compared with those from *X. tropicalis* (gene build v9.0, Xenbase.org, Aug. 2017).

Additional file 1: Figure S3: Scatterplot showing the coverage of each *R. marina* CDS length in base pairs compared with the corresponding CDS match in *X. tropicalis* (gene build v9.0, Xenbase.org, Aug. 2017). CDS length matches were extracted from BLASTx queries with *evalue = 10E-3* and *–max_target_seqs=1.*

Additional file 1: Figure S4: Venn diagram showing an overview of the significant BLASTx matches (e-value < 10^–3^) for our *R. marina* assembly against the proteins from *X. tropicalis, X. laevis*, and *N. parkeri*. Venn diagram build using the Venn diagram webserver: http://bioinformatics.psb.ugent.be/webtools/Venn/.

Additional file 1: Figure S5. Venn diagram showing an overview of the significant BLASTx matches (e-value < 10^–3^) for our *R. marina* transcripts with annotations (31 103) against proteins from *X. tropicalis, X. laevis*, and *N. parkeri*. Venn diagram built using the Venn diagram webserver: http://bioinformatics.psb.ugent.be/webtools/Venn/

Additional file 2: Table S1: Summary of OrthoFinder analysis. Gene build versions are given in the species heading where appropriate.

### Competing interests

We declare no competing interests.

### Abbreviations

BUSCO: Benchmarking universal single copy orthologs; bp = base pair; CDS: coding sequence; GO: Gene Ontology; SRA: Short Read Archive; TSA: Transcriptome Shotgun Assembly.

### Ethics statement

Ethics approval for the capture of wild Australian samples was provided under the University of Sydney permit 2014/562, the rearing of tadpoles by the University of Sydney permit 2013/6033, and Brazilian samples under the Brazilian Federal Chico Mendes Institute for Biodiversity Conservation (ICMBio), through license number 38 047–3.

### Author contributions

M.F.R., M.V., and L.A.R. collected animals and conducted the sample preparation. J.G.R. and M.R.C. conducted tadpole rearing. M.F.R., F.S., D.M.S., M.C., and L.A.R. conducted RNA isolation for sequencing and library construction. AJW contributed samples. M.F.R. conducted transcriptome assemblies and analysis. M.F.R., F.S., R.S., and L.A.R. wrote the manuscript and participated in study design. All authors commented on the manuscript and approved the final submission.

## Supplementary Material

GIGA-D-17-00098_Original-Submission.pdf

GIGA-D-17-00098_Revision-1.pdf

GIGA-D-17-00098_Revision-2.pdf

Response-to-Reviewer-Comments_Original-Submission.pdf

Response-to-Reviewer-Comments_Revision-1.pdf

Reviewer-1-Report-(Original-Submission) -- Taejoon Kwon 04 Jun 2017 Reviewed

Reviewer-1-Report-(Revision-1) -- Taejoon Kwon 04 Oct 2017 Reviewed

Reviewer-2-Report-(Original-Submission) -- John Malone 08 Jun 2017 Reviewed

Reviewer-2-Report-(Revision-1) -- John Malone 10 Oct 2017 Reviewed

Reviewer-3-Report-(Original-Submission) -- Masanori Taira 14 Jun 2017 Reviewed

Supplement Figures
