# Supplementary material for: Improving amphibian genomic resources: a multitissue reference transcriptome of an iconic invader
Source: Gigascience. 2017 Nov 27;7(1):gix114. doi: 10.1093/gigascience/gix114 (PMC5765561; doi:10.1093/gigascience/gix114)
Supplement: GIGA-D-17-00098_Revision-1.pdf [file gix114_giga-d-17-00098_revision-1.pdf]

GigaScience

Improving amphibian genomic resources: a multi-tissue reference transcriptome of an iconic invader

--Manuscript Draft--

**Manuscript Number:**	GIGA-D-17-00098R1
**Full Title:**	Improving amphibian genomic resources: a multi-tissue reference transcriptome of an iconic invader
**Article Type:**	Data Note
**Funding Information:**	Australian Research Council (FL120100074) — Prof Richard Shine<br>Australian Research Council (DE150101393) — Dr Lee A Rollins
**Abstract:**	Background<br>Cane toads (Rhinella marina) are an iconic invasive species introduced to four continents and well utilized for studies of rapid evolution in introduced environments. Despite the long introduction history of this species, its profound ecological impacts and its utility for demonstrating evolutionary principles, genetic information is sparse. Here we produce a de novo transcriptome spanning multiple tissues and life stages to enable investigation of the genetic basis of previously identified rapid phenotypic change over the introduced range.<br>Findings<br>Using approximately 1.9 billion reads, from developing tadpoles and 6 adult tissue-specific cDNA libraries, and a transcriptome assembly pipeline encompassing 100 separate de novo assemblies, we constructed 62,202 transcripts, of which we functionally annotated ~50 %. Our transcriptome assembly exhibits 90% full-length completeness of the BUSCO (benchmarking universal single-copy orthologs) dataset. Robust assembly metrics and comparisons to several available anuran transcriptomes and genomes indicate that our cane toad assembly is one of the most complete anuran genomic resources available.<br>Conclusion<br>This comprehensive anuran transcriptome will provide a valuable resource for investigation of genes under selection during invasion in cane toads, but will also greatly expand our general knowledge of anuran genomes, which are underrepresented in the literature. The dataset is publically available in NCBI and GigaDB to serve as a resource for other researchers.
**Corresponding Author:**	Mark F Richardson<br>Deakin University - Geelong Campus at Waurn Ponds<br>Waurn Ponds, VIC AUSTRALIA
**Corresponding Author Secondary Information:**	
**Corresponding Author's Institution:**	Deakin University - Geelong Campus at Waurn Ponds
**Corresponding Author's Secondary Institution:**	
**First Author:**	Mark F Richardson
**First Author Secondary Information:**	
**Order of Authors:**	Mark F Richardson<br>Fernando Sequeira<br>Daniel Selechnik<br>Miguel Carneiro<br>Marcelo Vallinoto<br>Jack G Reid

Powered by Editorial Manager® and ProduXion Manager® from Aries Systems Corporation

	Andrea J West
	Michael R Crossland
	Richard Shine
	Lee A Rollins
**Order of Authors Secondary Information:**	
**Response to Reviewers:**	Dear Dr Edmunds,<br><br>Thank you for the opportunity to submit a revised manuscript. We thank the reviewers for their positive and helpful comments that were valuable in revising and improving the manuscript. We have responded to all the reviewers' comments and incorporated changes into the revised manuscript.<br><br>Please find below our specific responses to the reviewer's comments and details of the changes carried out to the manuscript. We have also made additional effort within both the manuscript and associated data to conform to MINSEQE guidelines. As such, we have additional datasets we would like to include in GigaDB. Our lab protocols follow standard manufacturer protocols but we are happy to reproduce these in protocols.io if required.<br><br>We have uploaded a 'marked' manuscript will all changes shown in track changes (MS Word) and a clean version of the manuscript.<br><br>We confirm that this manuscript has not been published elsewhere and is not under consideration by another journal. All authors approved the manuscript and agree with its submission. We hope that our revised manuscript will meet the criteria needed for publication in Gigascience and we look forward to hearing from you.<br><br>Detailed response to reviewers:<br><br>NOTE: square brackets donate the line numbers corresponding to changes made in the track changes document.<br><br>Reviewer #1:<br><br>Richardson et al., reported the comprehensive transcriptome of cane toad Rhinella marina derived from the RNA-seq data of seven different tissues (some of them are from previously published data). Due to the estimated large genome size and limited availability of amphibian genomic resources, as the authors pointed out, I agree that this result would be useful resources in the community. However, there are some information missing to interpret their result.<br><br>(1) Although the authors described the detailed information about the samples, I cannot find the corresponding sample IDs in Table 1. Please provide the sampling location information and gender information in Table 1.<br><br>Response: This information has now been added to Table 1.<br><br>(2) Table 4 pointed out that this study provided 20-30% more transcriptome, compared to previously reported data (muscle & liver; 90% vs 60-69%). I wonder why. Is it because of brain samples, which may contain many neuro-specific genes? Or is it just general improvement of the data and/or analysis method? By discussing the function of "newly identified" genes in this study, I think the authors can infer the reason.<br><br>Response: We believe it is due to general improvement, 1) by the addition of more data, and 2) a more robust and thorough assembly methodology. If it was due to the addition of a single tissue (such as brain) we would expect to see many tissue-specific genes expressed. Figure S1;additional file 1 (as requested by reviewer 3) shows that this is not the case, with the largest set of tissue specific expression only accounting for ~5% (testes) of transcript expression (total tissue specific expression is < 7 %).<br><br>(3) How many X. tropicalis (or human) orthologs are identified? Reciprocal best hits

may not be good way to compare, because it is hard to reduce the isoforms in transcriptome data without a genome.

Response: We agree there are issues with reciprocal best hits as a method for identifying orthologues. As such, we have included a more thorough method (OrthoFinder) for identifying orthologues between the three available anuran genomes and our assembly. While there is still an issue with isoforms in the transcriptome data, the use of 'orthogroups' in this program allows for multiple isoforms to be included within an orthologous group – something not possible with the reciprocal best hit method. Using this method, we have identified 13,657 orthologues between R. marina and X. tropicalis and 4,586 that are found as single copy orthologues between all four species. More specifics are provided in the manuscript and we have made this analysis available in the associated public data repository. [293-313]

(4) It is not clear how the authors run BUSCO for comparison. For example, which version of X. laevis & X. tropicalis sequences are used?

Response: We have provided clarification of how this analysis was run [221-227] and given the versions of all gene builds used in the legend of Table 4 and the main text [226] .

Reviewer #2:

The study provides an expanded transcriptome resource for the marine toad, expanding genomic resources available for amphibians, which in general are underrepresented among genomic datasets. The study generated RNA-Seq datasets for brain, spleen, tadpoles, ovary, and testis in the marine toad and makes use of existing datasets for liver and muscle. The detailed methods documenting how samples were collected, and origin of samples were fantastic to read and should be a standard for others.

The authors use several assemblers to analyze the data, employing a variety of kmer sizes for some tools but not for Trinity. This is understandable for the version of Trinity used in the manuscript, however newer versions of Trinity permit changing kmer sizes. A newer version of Trinity might be useful for their analyses and help in development of their over-assembly. Arguably however, their use of multiple assemblers is likely very robust and was nice to read.

Response: Thank you for highlighting that new versions of Trinity now permit the changing of k-mers. As the reviewer states, because of the use of multiple assemblers (including those with the option to vary k-mers) and multiple parameter sets we feel that further inclusion of more assemblies into the over-assembly (which already contains 100 separate assemblies) is unlikely to provide a more robust final assembly.

In general, I found the manuscript to be appropriate as a data note as there is no question these data will be useful. The comparison to other anuran transcriptomes is somewhat limited given the many more amphibian transcriptomes datasets that currently exist. More tissues would be useful beyond the addition of brain, spleen, tadpole and gonad which does not expand the resource much and but should be a useful advance for others.

Response: While more separate tissues would be nice to include in the assembly it is currently beyond our means for this project. We have included whole tadpoles in the assembly, which does contain all tissues (albeit it during a developmental stage). We have also included a more detailed comparison to other anuran resources (please see specific responses to reviewer 1 and 3) and included a more thorough investigation of anuran orthologues [293-313].

Reviewer #3:

The read number of each sample is sufficient (more than 1 M), and the number of genes is reasonable, but the quality control of de novo assembly is insufficient.

	Major comments:<br>The authors need more quality controls as follows:<br>i) Histograms of lengths of mRNAs and CDSs compared with X. tropicalis.<br><br>Response: This is now provided as Figure S2 (additional file 1) and discussed in the main text [205-209].<br><br>ii) Coverage of the length of each CDS compared with that of X. tropicalis, using a scatter plot, for example.<br><br>Response: This is now provided as Figure S3 (additional file 1) and discussed in the main text [209-211].<br><br>iii) Comparison of all annotated transcripts with those of X. tropicalis, X. laevis, and Nanorana<br><br>Response: This is now provided as Figure S5 (additional file 1) and discussed in the main text [286-291].<br><br>iv) Blastx analysis of all transcripts for those of X. tropicalis and X. laevis.<br><br>Response: This is now provided as Figure S4 (additional file 1) and discussed in the main text [264-286].<br><br>v) Comparison of all transcripts between the 6 adult tissues and tadpoles of Rhinella marina (cane toad) to show the percentages of common and tissue-specific genes in each tissue using a Venn diagram.<br><br>Response: We have been unable to construct a meaningful Venn/Euler diagram which shows all of the combinations between our 7 tissues that would be interpretable. We agree with the reviewer that this is an important comparison to include, so we have produced a simpler schematic showing the percentage of genes common to all tissues and the percentage unique to each tissue (as per the reviewer's main request). Additionally, we have included a text file which contains the full set of comparisons in the associated data repository. We present this data in brief in the main text and included the figure in the supplement as Figure S1(additional file 1) [161-163].<br><br>Minor comments<br><br>Table 4: Either Table 4 or its legend needs to be revised, because laevis (g) is Session et al. (5); and tropicalis (h) is Hellsten et al. (4). Which one is correct, the table, legend, or reference?<br><br>Response: Corrected – the legend citation was incorrect and has been revised.<br><br>If X. laevis is not mislabeled, the "Complete" (61%) seems to be too low compared to X. tropicalis (92%). Because X. laevis is allotetraploid, how did the authors calculate the completeness? Did the authors use the latest version of X. laevis gene models?<br><br>Response: We have updated this analysis to include the latest (as of 30th August 2017 - http://www.xenbase.org/other/static/ftpDatafiles.jsp) gene models for both Xenopus species and they both now show comparable results [Table 4, 237]. Completeness is calculated using the BUSCO pipeline and we have made this more explicit in the text – "Complete" refers to those BUSCOs that have a full-length match within the assembly. [228, 249-253]<br><br>The "Duplicated" is 59% (X. laevis) or 62% (X. tropicalis), which are much higher than the others. How is it so? The definition of these two terms (Complete and Duplicated) and others must be clarified and explained in the manuscript.<br><br>Response: We point the reviewers to the new results of this analysis, which show lower levels of complete BUSCOs that are duplicated. We also clarify these terms in the manuscript and provide standalone definitions in the Table legend for clarity. [230-232, Table 4]

Powered by Editorial Manager® and ProduXion Manager® from Aries Systems Corporation

Additional Information:	
**Question**	**Response**
Are you submitting this manuscript to a special series or article collection?	No
**Experimental design and statistics**<br><br>Full details of the experimental design and statistical methods used should be given in the Methods section, as detailed in our Minimum Standards Reporting Checklist. Information essential to interpreting the data presented should be made available in the figure legends.<br><br>Have you included all the information requested in your manuscript?	Yes
**Resources**<br><br>A description of all resources used, including antibodies, cell lines, animals and software tools, with enough information to allow them to be uniquely identified, should be included in the Methods section. Authors are strongly encouraged to cite Research Resource Identifiers (RRIDs) for antibodies, model organisms and tools, where possible.<br><br>Have you included the information requested as detailed in our Minimum Standards Reporting Checklist?	Yes
**Availability of data and materials**<br><br>All datasets and code on which the conclusions of the paper rely must be either included in your submission or deposited in publicly available repositories (where available and ethically appropriate), referencing such data using a unique identifier in the references and in the "Availability of Data and Materials" section of your manuscript.<br><br>Have you have met the above requirement as detailed in our Minimum Standards Reporting Checklist?	Yes

Manuscript

Click here to download Manuscript Richardson_etal_manuscript_revised.docx

Improving amphibian genomic resources: a multi-tissue reference transcriptome of an iconic invader

Mark F. Richardson[1,2], Fernando Sequeira[3], Daniel Selechnik[6], Miguel Carneiro[3,4], Marcelo Vallinoto[5], Jack G. Reid[2], Andrea J. West[2], Michael R. Crossland[6], Richard Shine[6] and Lee A. Rollins[2]

[1] Deakin University, Bioinformatics Core Research Group, 75 Pigdons Road, Locked Bag 20000, Geelong, VIC 3220, Australia

[2] Deakin University, School of Life and Environmental Sciences, Centre for Integrative Ecology (Waurn Ponds Campus), 75 Pigdons Road, Locked Bag 20000, Geelong, VIC 3220, Australia

[3] CIBIO-InBIO, Centro de Investigação em Biodiversidade e Recursos Genéticos, Campus Agrário de Vairão, Universidade do Porto, 4485-661, Vairão, Portugal

[4] Departamento de Biologia, Faculdade de Ciências, Universidade do Porto, Rua do Campo Alegre s⁄n., 4169-007 Porto, Portugal

[5] Laboratório de Evolução (LEVO), Instituto de Estudos Costeiros (IECOS), Universidade Federal do Pará, Campus de Bragança, Pará, Brasil

[6] School of Life and Environmental Sciences, The University of Sydney, NSW 2006, Australia

Keywords: *de novo* assembly, *Bufo marinus*, cane toad, *Rhinella marina*, invasive species, RNA-Seq, transcriptome, anuran, amphibian

Abstract

Background

Cane toads (*Rhinella marina*) are an iconic invasive species introduced to four continents and well utilized for studies of rapid evolution in introduced environments. Despite the long introduction history of this species, its profound ecological impacts and its utility for demonstrating evolutionary principles, genetic information is sparse. Here we produce a *de novo* transcriptome spanning multiple tissues and life stages to enable investigation of the genetic basis of previously identified rapid phenotypic change over the introduced range.

Findings

Using approximately 1.9 billion reads, from developing tadpoles and 6 adult tissue-specific cDNA libraries, and a transcriptome assembly pipeline encompassing 100 separate *de novo* assemblies, we constructed 62,202 transcripts, of which we functionally annotated ~50 %. Our transcriptome assembly exhibits 90% full-length completeness of the BUSCO (benchmarking universal single-copy orthologs) dataset. Robust assembly metrics and comparisons to several available anuran transcriptomes and genomes indicate that our cane toad assembly is one of the most complete anuran genomic resources available.

Conclusion

This comprehensive anuran transcriptome will provide a valuable resource for investigation of genes under selection during invasion in cane toads, but will also greatly expand our general knowledge of anuran genomes, which are underrepresented in the literature. The dataset is publically available in NCBI and GigaDB to serve as a resource for other researchers.

Data Description

Background

It is well established that genome size across taxa is related to repetitive DNA content [1]. Highly repetitive genomes present significant challenges to genome assembly [2], which likely accounts for the scarcity of large genome sequences currently available. Anuran genome size is highly variable (C-values of 0.95-13.02; [3]) and, to date, genome sequences of only three anurans have been published: *Xenopus tropicalis* [4], *X. laevis* [5] and *Nanorana parkeri* [6]. Large genomes typify many Bufonids, including the cane toad (*Rhinella marina*; average reported C-value = 4.79 [3]) and none have been sequenced to date. Transcriptome sequencing provides a tenable alternative to genome sequencing in anurans because the large, repetitive, non-coding regions typical of their large genomes are not sequenced [7].

Cane toads are an excellent model for the study of invasion. Because they were intentionally and repeatedly introduced to novel environments as a biocontrol agent, their introduction history is well-documented [8]. A wealth of evolutionary and ecological knowledge about cane toads currently exists, documenting phenotypic evidence of rapid evolution in introduced environments, but genomic data are scarce [9]. Providing access to well-developed genomic resources for the cane toad will enable the investigation of the genetic basis of traits underlying invasion ability in this species, which will in turn significantly advance our understanding of invasion genetics for all species. Here we present a *de novo* transcriptome assembly covering multiple *R. marina* tissues and life stages, representing one of most complete anuran genomic resources reported to date.

Samples

Cane toad samples and tissues (seven in total) used in this study were obtained from several sources within the invasive (Australian) and native (Brazilian) range. Several different methods were used to prepare and sequence samples; for simplicity we describe the samples and data sources used based on the tissue types sequenced (Table 1). *Brain and Spleen* – four adult female toads were collected across two sites in Australia, two from Durack (15.9419° S, 127.2202° E), Western Australia, and two from Gordonvale (17.0972° S, 145.7792° E), Queensland, in May 2015. Toads were euthanized using lethal injection of 150mg/kg sodium pentobarbital and whole brain and spleen was harvested and immediately stored in RNAlater (Qiagen, USA), kept at 4 °C, then transferred to -80 °C for storage until RNA extraction. *Tadpoles* – We conducted a tadpole rearing experiment in March 2015. Four adult toads (2 males and 2 females) were collected from both Oombulgurri (15.1818° S, 127.8413° E), Western Australia, Australia and Innisfail (17.4963° S, 146.0465° E), Queensland, Australia. To obtain egg clutches, pairs of adult male and female toads per population (i.e. 2 separate male x female crosses) were subcutaneously injected with 0.25mg/ml Leuproelin acetate (Lucrin® Abbott Australasia, Kurnell, Australia) in amphibian Ringer's solution to stimulate spawning; males received 0.25 ml and females 0.75 ml. The pairs of male and female toads were left overnight in 750L plastic enclosures, containing bore water, to lay and fertilize egg clutches. Egg clutches were removed and placed in 17L tanks containing continuously aerated bore water and monitored to ensure fertilization had occurred. Embryos were selected once they reached Gosner stage 16-17 [10]. Three replicates of five fertilised embryos were removed per clutch and placed in 1L containers, each with 750ml bore water, where they were raised until 10 days old; water was changed daily and developing tadpoles were fed 12mg of a commercial algae supplement (Hikari algae pellets, Kyorin, Himeji City, Japan) after each water change. One tadpole from each of the three replicate tadpole tanks per clutch

was euthanized (immersion in 2g/L Tricaine methanesulfonate) and immediately stored in RNAlater, kept at 4 °C, then transferred to -80 °C for storage until RNA extraction.

Table 1. Cane toad samples used to generate the de novo reference transcriptome

Tissue	Origin	Platform	Sample ID (library size)	Sampling location	Sex	SRA
Brain	Australia	HiSeq 2500 (2x125 bp)	B19 (23.9 M)	Durack	F	SRR5446736
			B20 (27.7 M)	Durack	F	SRR5446735
			B31 (24.8 M)	Gordonvale	F	SRR5446734
			B32 (22.3 M)	Gordonvale	F	SRR5446733
Spleen	Australia	HiSeq 2500 (2x125 bp)	S1 (23.8 M)	Gordonvale	F	SRR5446732
			S2 (25.0 M)	Gordonvale	F	SRR5446732
			S18 (24.7 M)	Durack	F	SRR5446732
			S19 (23.6 M)	Durack	F	SRR5446732
Muscle	Australia	HiSeq 2000 (2x100 bp)	RM0021M (93.8 M)	El Questro	F	SRR1910534 SRR1910535
			RM0094M (88.2 M)	Purnululu	F	SRR1910543
			RM0108M (97.6 M)	Innisfail	F	SRR1910545
			RM0169M (80.0 M)	Rossville	F	SRR1910549
Tadpole	Australia	HiSeq 2500 (2x125 bp)	T1 (26.4 M)	Oombulgurri	Both	SRR5446728
			T4 (24.5 M)	Oombulgurri	Both	SRR5446727
			T7 (23.2 M)	Innisfail	Both	SRR5446726
			T10 (25.7 M)	Innisfail	Both	SRR5446725
Liver	Brazil	HiSeq 2000 (2x75bp)	RMTP (536.8 M)	Macapá city	NA	SRR1514601
Ovary	Brazil	HiSeq 1500 (2x125 bp)	AR19 (434.1 M)	Macapá city	F	SRR5446724
Testes	Brazil	HiSeq 1500 (2x125 bp)	AR05 (410.5 M)	Macapá city	M	SRR5446723

Library size is given as raw sequenced reads in millions (M), sex donated as F - female, M – male, Both – sample contains mixed individuals of both sexes, NA – information unknown.

Total RNA was extracted from each of the brain, spleen and tadpole samples using Qiagen RNeasy kits (Qiagen, USA), following the manufacturers protocol with an additional DNase

digestion step. Extracted RNA was quantified using a Quibit RNA HS assay on a Qubit 3.0 Fluorometer (Life technologies, USA). For the tadpole sequencing, total RNA from the three 'replicate' tadpoles per clutch were pooled in equal quantities, resulting in 4 pooled samples. Two µg of total RNA per sample were sent to Macrogen (Macrogen Inc., Seoul, ROK), where mRNA libraries were constructed using the TruSeq mRNA v2 sample kit (Illumina Inc, San Deigo, USA) that included a 300 bp size selection step. Libraries were sequenced on one lane of Illumina HiSeq 2500 (Illumina Inc, San Deigo, USA), generating 295.6 million paired-end 2 x 125bp reads. Raw reads are available in the NCBI Short Read Archive (SRA) under the Bioproject Accession: PRJNA382870.

Muscle – We downloaded raw fastq files (NCBI Bioproject Accession: PRJNA277985; paired-end 2 x 100bp; Illumina HiSeq-2000) for four adult female toads (RM0021M, RM0094M, RM0108M and RM0169M) across four populations in Australia (El Questro, 16.007872S, 128.020494E; Purnululu National Park, 17.4334° S, 128.3018° E, both Western Australia; Innisfail, 17.4963° S, 146.0465° E; Rossville, 15.7054° S, 145.2229° E, both Queensland) previously used to build a *de novo* muscle (*triceps femoris*) transcriptome [9].

Ovary and Testes – Two adult toads (1 male and 1 female) were collected from Macapá city (0.0432° S, 51.1241° W), Amapá state, Brazil in December 2015. Toads were euthanized as described above and ovary and testes were excised and immediately stored in RNAlater then kept at 4 °C for storage until RNA extraction. Total RNA was extracted using Qiagen RNeasy kits, following the manufacturers protocol with an additional DNase digestion step. Extracted RNA was quantified using a Qubit RNA BR assay and RNA integrity (RIN) was assessed using a Tapestation 2200 (Aligent Tech., Santa Clara, CA, USA) with an RNA Screen. One µg of total RNA per sample was used to construct mRNA libraries using the TruSeq mRNA

v2 sample kit, which included a 130–350 bp size selection step. Both libraries were run on a HiSeq 1500 using Illumina V4 PE chemistry across two lanes (one lane for each sample) generating 844.6 million paired-end 2 x 125bp reads. Raw reads are available in the NCBI SRA under the Bioproject Accession: PRJNA382870.

Liver – We downloaded raw fastq files (NCBI Bioproject Accession: PRJNA255079; paired-end 2 x 75 bp; Illumina HiSeq-2000) from a pool of five adult toads from Macapá city, Amapá state, Brazil, previously used to build a *de novo* liver transcriptome [11].

Data preprocessing and multiple *de novo* transcriptome assemblies

Raw reads from each sample were first processed with Trimmomatic v0.33 [12], using the following parameters: ILLUMINACLIP:TruSeq3-PE.fa:2:30:10:4 HEADCROP:13 AVGQUAL:30 MINLEN:36, to: *i*) remove adaptor sequences, *ii*) trim the first 13 bp of a read, *iii*) discard reads with an average quality < Phred 30 and *iv*) remove reads < 36bp after processing. We then concatenated reads into two input data sets, one containing all samples from Australia and one containing those from Brazil. To reduce the computational load of assembly we used the *in silico* normalization approach implemented in Trinity v2.1.1 [13], with --*normalize_max_read_cov=50,* on both of the input data sets. The normalized Australia and Brazil data sets contained ~42.2 million and ~82.2 million reads, respectively. Multiple independent *de novo* transcriptome assemblies were conducted for each of the input data sets, resulting in 100 separate assemblies (see Table 2). In brief, we used three assemblers: Trinity, with default parameters and --*min_contig_length=300*; SOAPdenovo-Trans v1.03 [14], with 13 different k-mers (apart from the Brazil input set which had 12) for each combination of *EdgeCovCutoff=2*, *mergeLevel=1* and *EdgeCovCutoff=3*, *mergeLevel=2* - the parameters -f, -F and *minContigLen=200* were the same for all assemblies; velvet v1.2.09/oases v0.2.08

[15,16], with 12 different k-mers for each combination of *-cov_cutoff=3*, *-min_pair_count=4* and *-cov_cutoff=5*, *-min_pair_count=6*, where *-ins_length=300* and *-min_trans_lgth=200* were consistent across assemblies. The individual assemblies were then compiled into an 'over-assembly' of ~ 42 million transcripts. To reduce redundancy in the 'over-assembly' we used the tr2aacds pipeline from the Evidential Gene package [17], which selects the 'optimal' set of transcripts based on their coding potential. This reduced the redundant 'over-assembly' to the final assembly of 62,202 transcripts. Of these, 50 % (31,040) are commonly expressed among the seven different tissues used in the assembly, while a total of 6.64 % exhibit tissue specific expression (see Fig. S1: Additional file 1. and [32]). We then used TransDecoder v3.0.0 to predict protein coding sequences (CDS) with a minimum CDS of 100bp. Transvestigator [18] was used to prepare the final assembly for submission to NCBI's Transcriptome Shotgun Assembly (TSA) database – accessible through the PRJNA383966 accession. Results from the assembly pipeline are described in Table 3. As the 'dropset' – those transcripts not kept in the 'optimal' tr2aacds output - may contain other biologically relevant transcripts, such as non-coding RNAs and active transposable elements, we also provide these transcripts in the associated GigaDB repository [32].

Table 2. *De novo* assembler parameters used to produce the 'over-assembly'

Assembler	*k*-mers	Parameter combinations	No. of assemblies
Trinity	25	Default	Aus 1, Brazil 1
SOAPdenovo-Trans	21, 25, 29, 33, 37, 41, 45, 49, 59, 69, 79, 89, 99 (No 99 for the Brazil input set)	*EdgeCovCutoff=2* and *mergeLevel=1;* *EdgeCovCutoff=3* and *mergeLevel=2*	Aus 13, Brazil 12; Aus 13, Brazil 12
Velvet/Oases	21, 25, 29, 33, 37, 41, 45, 49, 59, 69, 79, 89	*cov_cutoff=3* and *min_pair_count=4;* *cov_cutoff=5* and *min_pair_count=6*	Aus 12, Brazil 12; Aus 12, Brazil 12
			Total: 100

Annotation

We conducted functional annotation based on our predicted protein sequences utilizing the automated Trinotate pipeline. Transcripts were first annotated based on sequence homology, where assembled nucleotides and translated CDS sequences were used in BLASTx and BLASTp searches, against the UniProt/Swiss-Prot database (downloaded Feb. 2017) using a standalone version of blast v 2.2.26+ [19], with an e-value cutoff of $1x\ 10^{-5}$. Pfam [20] functional domains (downloaded Feb. 2017) were identified in protein coding sequences using hmmscan [21]; signal peptides and transmembrane domains were assigned using hidden Markov model prediction implemented in SignalP v4.1 [22] and TMHMM v2.0c [23] respectively. Finally, transcripts were compared to curated annotations in eggNOG [24] and Gene Ontology (GO) [25] databases. A summary of annotation metrics is provided in Table 3. The combined Trinotate functional annotations to the TSA assembly are available in the associated GigaDB, see [32].

Table 3. Summary of transcriptome assembly and annotation statistics compared to previous cane toad transcriptomes

	This study	Muscle[a]	Liver[b]
Assembly			
Filtered read pairs	945,348,780	99,462,214	265,684,605
in silico normalized reads	129,051,008	18,713,526	-
Assembly size (bp)	83,724,193	60,388,685	80,251,892
Number of transcripts	62,202	57,580	131,020
N50	2,377	1,871	916
Average length (bp)	1,346	1,049	613
Minimum length (bp)	297	201	201
Maximum length (bp)	99,438	40,546	17,369
Median length (bp)	698	577	331
GC %	46.05	45.06	44.32

Transcripts with CDS	62,202	19,751	-
Annotation			
Transcripts with BLASTx hit	31,103	21,533	-
Transcripts with BLASTp hit	28,560	16,754	-
Transcripts with GO terms	28,399	19,500	-

[a][9];[b][11]

Quality and completeness of the cane toad transcriptome

To evaluate our new multi-tissue transcriptome assembly, we used three comparative approaches to assess relative quality and completeness. First, we compared core assembly statistics of the new assembly to our two previous cane toad single-tissue transcriptomes derived from muscle and liver tissue (see Table 3). The inclusion of data from multiple tissues (encompassing a 9.5- and 3.5-fold increase in read input compared to the muscle and liver transcriptomes, respectively) resulted in increases of all assembly metrics, apart from the number of assembled transcripts, which fell compared to the liver transcriptome (Table 3). Notably, mean transcript length increased from 613 (liver) to 1,346 bp and transcript n50 increased from 916 (liver) to 2,377 bp. The total assembled bases were similar between the multi-tissue transcriptome and that assembled from liver, yet higher (~20 Million bp) than that produced from muscle tissue. Additionally, our assembly exhibits comparable lengths of mRNAs and CDS to those from *X. tropicalis* (gene build v9.0, downloaded Xenbase.org, Aug. 2017), see (Fig. S2: Additional file 1.), albeit with a greater frequency of shorter features. This is not unexpected given we are comparing a *de novo* transcriptome assembly to gene models from a genome assembly. Also, our assembly shows good coverage of the lengths of CDS features compared to *X. tropicalis* (Fig. S3: Additional file 1.), given both species are substantially divergent. Importantly, the new *R. marina* multi-tissue assembly increases the coverage of transcripts containing protein coding sequences with associated

Blast matches and Gene Ontology annotations compared to the previously available *R. marina* assemblies.

Second, we evaluated the new assembly using the BUSCO (benchmarking universal single-copy orthologs) vertebrate gene set [26], which uses 3023 near-universal orthologs (hereafter BUSCOs) to evaluate relative completeness of assemblies and compared the results to the previous *R. marina* assemblies and those of several available amphibian transcriptomes and genomes (see Table 4.). We used BUSCO v1.2 [26] with the default e-value cutoff of 0.01 and *–mode=Trans* for all the transcriptomes compared and *–mode=OGS* for the genome comparisons (using the *N. parkeri* v2.0, *X. tropicalis* v9.0 and *X. laevis* v9.1_1.8.3.2 gene builds downloaded from Xenbase.org, Aug. 2017). Our multi-tissue assembly had a much higher percentage of complete BUSCOs (90 %), apart from the two *Xenopus* genomes which exhibited comparable results (*X. tropicalis*, 91 % and *X. laevis*, 97 %). Additionally, our multi-tissue transcriptome has low BUSCO missingness, intermediate duplication of complete BUSCOs and the second lowest level of fragmented BUSCOs. In contrast to the previous *R. marina* transcriptomes specifically, the new assembly has less fragmented and 20 – 30 % more complete BUSCO genes – suggesting the presence of more full-length transcripts. Overall the comparison of BUSCO results revealed our assembly to be one of the most complete references available for anurans.

Table 4. BUSCO analysis of transcriptome completeness

	Complete BUSCOs (%)	Complete and duplicated BUSCOs (%)	Fragmented BUSCOs (%)	Missing BUSCOs (%)
R. marina transcriptomes				
This study	90	4.7	1.7	7.8
Muscle[a]	60	4.6	5.7	33

Liver[b]	69	0.6	4.1	26
Select anuran transcriptomes				
Bufotes viridis[c]	26	0.3	15	57
Rana catesbeiana[d]	79	42	2.8	17
Pelohylax nigromaculatus[e]	50	0.4	7.8	41
Microhyla fissipes[f]	73	1.2	4.7	21
Select anuran genomes				
Xenopus laevis[g]	97	51	1.4	1.4
Xenopus tropicalis[h]	91	4.1	3.7	4.9
Nanorana parkeri[i]	76	2.8	9.0	14

[a][9],[b][11],[c][7],[d][27],[e][28],[f][29],[g][5],[h][4], [i][6] Complete BUSCOs refer to those with a full-length match in the assembly; Complete and duplicated refers to those BUSCOs that are complete within an assembly but have multiple matches present; Fragmented are those BUSCOs that only have a partial match in the assembly and Missing refers to those BUSCOs that do not have a corresponding match in the assembly.

Third, we compared the multi-tissue transcriptome to the previous cane toad transcriptomes and the three currently available Anuran genomes through both standard and reciprocal best-hit BLAST approaches. The standard approach revealed 40,741 (65.5 %) and 31,189 (50.1 %) of our new assembly had significant matches (e-value $<10^{-3}$) to the liver and muscle transcriptomes, respectively. The reciprocal best-hit approach reduced the number of significant matches to both the liver (23,943; 38.5 %) and muscle (15,892; 25.5%) transcriptomes, which may in part be due to transcripts mapping to multiple isoforms in the different assemblies. This, together with the high number of protein-coding transcripts in the multi-tissue assembly, indicates the new assembly still contains some redundancy and that we have assembled multiple transcripts variants for some genes. Standard BLAST comparisons of our assembly to the *X. tropicalis*, *X. laevis* and *N. parkeri* proteins exhibited 40,275 (64.7 %), 40,218 (64.7 %) and 40,244 (64.7 %) significant matches, respectively; 37,064 of our assembled transcripts with significant matches are common to all three species (Fig. S4:

Additional file 1.). Of our 31,103 assembled transcripts with annotations, 97.8 % (30,423) have significant matches to *X. tropicalis,* while 97.9 % (30,465) have matches to *X. laevis* and 98.3 % (30,574) to *N. parkeri*; 96.3 % (29,967) are common to all three species (Fig. S5: Additional file 1.). The high percentage of annotated transcripts with matches to *X. tropicalis, X. laevis* and *N. parkeri* provides further evidence that our assembly pipeline produced transcripts with strong anuran homology.

Identification of Anuran Orthologues

Current efforts to identify amphibian-specific genes has been hampered by a lack of high quality full-length genes for numerous amphibian species [30]. So far, the identification of amphibian-specific genes has not been possible as orthologous counterparts have only been identified between the two *Xenopus* genomes. We used OrthoFinder v1.1.10 [31] with default parameters to identify orthologues between our newly assembled *R. marina* CDS containing transcripts and the proteomes from *N. parkeri*, *X. tropicalis* and *X. laevis* (using the same gene build versions as in the BUSCO analysis). OrthoFinder identifies 'orthogroups' (a group of genes descended from a single gene in the last common ancestor of a group of species [31]) and then orthologues between each pair of species in the comparison. Because OrthoFinder classifies genes with multiple orthologues (i.e. many to many relationships) in 'orthogroups' it may reduce the impact multiple isoforms of the same gene have in such analyses. We assigned 60.2 % (94,516) of all the genes examined to 18,776 'orthogroups' (see Additional file 2.), of which 50% of all genes were found in 'orthogroups' with four or more genes. Additionally, we identified 12,674 'orthogroups' which contained genes from all four species and 4,586 of these consisted entirely of single-copy genes. The dataset presented here may be

useful for further research into identifying amphibian-specific genes, so we have included this analysis in its entirety in the associated GigaDB repository [32].

Conclusions

This comprehensive anuran transcriptome will not only serve as a valuable reference for investigation of genes under selection during invasion in cane toads, but will also expand our general knowledge of anuran genomes. Additionally, we have identified numerous orthologous transcripts to *X. tropicalis*, *X. laevis* and *N. parkeri* proteins, which may aid the identification of amphibian-specific genes – an important objective of AmphiBase [30].

Abbreviations

SRA: Short Read Archive; CDS: coding sequence; TSA: Transcriptome Shotgun Assembly; GO: Gene Ontology; BUSCO: Benchmarking universal single copy orthologs

Availability of supporting data

The datasets supporting the results presented here are available in the associated GigaDB repository [31]. All raw sequencing data used in this study is available in the SRA and associated with the following BioProject accessions: PRJNA277985, PRJNA255079, PRJNA382870 and PRJNA383966. The final transcriptome assembly has been deposited at DDBJ/EMBL/GenBank under the accession GFMT00000000. The version described in this paper is the first version, GFMT01000000.

Declarations

Acknowledgements

We thank Serena Lam and Chris Jolly for assistance with sample collection. This project was funded through: An Australian Research Council (ARC) Discovery Early Career Research Award (DE150101393) to LAR, ARC Laureate Fellowship to RS (FL120100074), Centre for Integrative Ecology research funds, Deakin University to MFR, FCT Investigator grant to MC [IF/00283/2014/CP1256/CT0012], Research project and research fellowship (PQ 10/2012 and Universal 14-2013) and post-doctoral fellowship to MV (232916/2013-6, CNPq) and FEDER (COMPETE, POCI-01-0145-FEDER-006821) and FCT (UID/BIA/50027/2013) funds to FS.

Competing interests

We declare no competing interests.

Ethics statement

Ethics approval for the capture of wild Australian samples was provided under the University of Sydney permit 2014/562, the rearing of tadpoles by the University of Sydney permit 2013/6033 and Brazilian samples under the Brazilian Federal Chico Mendes Institute for Biodiversity Conservation (ICMBio), through license number 38047-3.

Author contributions

MFR, MV and LAR collected animals and conducted the sample preparation. JGR and MRC conducted tadpole rearing. MFR, FS, DMS, MC and LAR conducted RNA isolation for sequencing and library construction. AJW contributed samples. MFR conducted

transcriptome assemblies and analysis. MFR, FS, RS and LAR wrote the manuscript and participated in study design. All authors commented on the manuscript and approved the final submission.

References

1. Kidwell MG. Transposable elements and the evolution of genome size in eukaryotes. Genetica. 2002;115:49–63.

2. Treangen TJ, Salzberg SL. Repetitive DNA and next-generation sequencing: computational challenges and solutions. Nat. Rev. Genet. 2011;13:36–46.

3. Gregory T. Animal Genome Size Database. 2015 http://www.genomesize.com Accessed 3 March 2017.

4. Hellsten U, Harland RM, Gilchrist MJ, Hendrix D, Jurka J, Kapitonov V, et al. The Genome of the Western Clawed Frog Xenopus tropicalis. Science. 2010;328:633–6.

5. Session AM, Uno Y, Kwon T, Chapman JA, Toyoda A, Takahashi S, et al. Genome evolution in the allotetraploid frog Xenopus laevis. Nature. 2016;538:336–43.

6. Sun Y-B, Xiong Z-J, Xiang X-Y, Liu S-P, Zhou W-W, Tu X-L, et al. Whole-genome sequence of the Tibetan frog Nanorana parkeri and the comparative evolution of tetrapod genomes. Proc. Natl. Acad. Sci. U. S. A. 2015;112:E1257-62.

7. Gerchen JF, Reichert SJ, Röhr JT, Dieterich C, Kloas W, Stöck M. A Single Transcriptome of a Green Toad (Bufo viridis) Yields Candidate Genes for Sex Determination and -Differentiation and Non-Anonymous Population Genetic Markers. PLoS One. 2016;11:e0156419.

8. Kraus F. Alien reptiles and amphibians : a scientific compendium and analysis. Springer; 2009.

9. Rollins LA, Richardson MF, Shine R. A genetic perspective on rapid evolution in cane toads (*Rhinella marina*). Mol. Ecol. 2015;24:2264–76.

10. Gosner KL. A Simplified Table for Staging Anuran Embryos and Larvae with Notes on Identification. Herpetologica. 1960;16:183–90.

11. Arthofer W, Banbury BL, Carneiro M, Cicconardi F, Duda TF, Harris RB, et al. Genomic Resources Notes Accepted 1 August 2014-30 September 2014. Mol. Ecol. Resour. 2015;15:228–9.

12. Bolger AM, Lohse M, Usadel B. Trimmomatic: a flexible trimmer for Illumina sequence data. Bioinformatics. 2014;30:2114–20.

13. Grabherr MG, Haas BJ, Yassour M, Levin JZ, Thompson DA, Amit I, et al. Full-length transcriptome assembly from RNA-Seq data without a reference genome. Nat. Biotechnol. 2011;29:644–52.

14. Xie Y, Wu G, Tang J, Luo R, Patterson J, Liu S, et al. SOAPdenovo-Trans: de novo transcriptome assembly with short RNA-Seq reads. Bioinformatics. 2014;30:1660–6.

15. Zerbino DR, Birney E. Velvet: algorithms for de novo short read assembly using de Bruijn graphs. Genome Res. 2008;18:821–9.

16. Schulz MH, Zerbino DR, Vingron M, Birney E. Oases: robust de novo RNA-seq assembly across the dynamic range of expression levels. Bioinformatics. 2012;28:1086–92.

17. Gilbert D. Accurate & complete gene construction with EvidentialGene. F1000Research. 2016;5.

18. DeRego T, Hall B, Ben-guin, Geib S. Transvestigator early release. Zenodo. 2014.

19. Camacho C, Coulouris G, Avagyan V, Ma N, Papadopoulos J, Bealer K, et al. BLAST+: architecture and applications. BMC Bioinformatics. 2009;10:421.

20. Finn RD, Bateman A, Clements J, Coggill P, Eberhardt RY, Eddy SR, et al. Pfam: the protein families database. Nucleic Acids Res. 2014;42:D222-30.

21. Eddy SR, Crooks G, Green R, Brenner S, Altschul S. Accelerated Profile HMM Searches. Pearson WR, editor. PLoS Comput. Biol. 2011;7:e1002195.

22. Petersen TN, Brunak S, von Heijne G, Nielsen H. SignalP 4.0: discriminating signal peptides from transmembrane regions. Nat. Methods. 2011;8:785–6.

23. Krogh A, Larsson B, von Heijne G, Sonnhammer EL. Predicting transmembrane protein topology with a hidden Markov model: application to complete genomes. J. Mol. Biol. 2001;305:567–80.

24. Powell S, Szklarczyk D, Trachana K, Roth A, Kuhn M, Muller J, et al. eggNOG v3.0: orthologous groups covering 1133 organisms at 41 different taxonomic ranges. Nucleic Acids Res. 2012;40:D284–9.

25. Ashburner M, Ball CA, Blake JA, Botstein D, Butler H, Cherry JM, et al. Gene Ontology: tool for the unification of biology. Nat. Genet. 2000;25:25–9.

26. Simão FA, Waterhouse RM, Ioannidis P, Kriventseva E V., Zdobnov EM. BUSCO: assessing genome assembly and annotation completeness with single-copy orthologs. Bioinformatics 2015;31:3210–2.

27. Birol I, Behsaz B, Hammond SA, Kucuk E, Veldhoen N, Helbing CC. De novo

Transcriptome Assemblies of Rana (Lithobates) catesbeiana and Xenopus laevis Tadpole Livers for Comparative Genomics without Reference Genomes. Plateroti M, editor. PLoS One 2015;10:e0130720.

28. Huang L, Li J, Anboukaria H, Luo Z, Zhao M, Wu H. Comparative transcriptome analyses of seven anurans reveal functions and adaptations of amphibian skin. Sci. Rep. 2016;6:24069.

29. Zhao L, Liu L, Wang S, Wang H, Jiang J. Transcriptome profiles of metamorphosis in the ornamented pygmy frog Microhyla fissipes clarify the functions of thyroid hormone receptors in metamorphosis. Sci. Rep. 2016;6:27310.

30. Kwon T. AmphiBase: A new genomic resource for non-model amphibian species. genesis. 2017;55:e23010.

31. Emms DM, Kelly S. OrthoFinder: solving fundamental biases in whole genome comparisons dramatically improves orthogroup inference accuracy. Genome Biology 2015;16:157

32. Richardson MF, Sequeira F, Sclechnik D, Carneiro M, Vallinoto M, Reid JG, et al. Supporting data for "Improving amphibian genomic resources: a multi-tissue reference transcriptome of an iconic invader" GigaDB doi XXX

Manuscript_track_changes
Click here to download Manuscript Richardson_etal_manuscript_gigascience_track_changes.docx

Improving amphibian genomic resources: a multi-tissue reference transcriptome of an iconic invader

Mark F. Richardson[1,2], Fernando Sequeira[3], Daniel Selechnik[6], Miguel Carneiro[3,4], Marcelo Vallinoto[5], Jack G. Reid[2], Andrea J. West[2], Michael R. Crossland[6], Richard Shine[6] and Lee A. Rollins[2]

[1] Deakin University, Bioinformatics Core Research Group, 75 Pigdons Road, Locked Bag 20000, Geelong, VIC 3220, Australia

[2] Deakin University, School of Life and Environmental Sciences, Centre for Integrative Ecology (Waurn Ponds Campus), 75 Pigdons Road, Locked Bag 20000, Geelong, VIC 3220, Australia

[3] CIBIO-InBIO, Centro de Investigação em Biodiversidade e Recursos Genéticos, Campus Agrário de Vairão, Universidade do Porto, 4485-661, Vairão, Portugal

[4]Departamento de Biologia, Faculdade de Ciências, Universidade do Porto, Rua do Campo Alegre s⁄n., 4169-007 Porto, Portugal

[5]Laboratório de Evolução (LEVO), Instituto de Estudos Costeiros (IECOS), Universidade Federal do Pará, Campus de Bragança, Pará, Brasil

[6] School of Life and Environmental Sciences, The University of Sydney, NSW 2006, Australia

Keywords: *de novo* assembly, *Bufo marinus*, cane toad, *Rhinella marina*, invasive species, RNA-Seq, transcriptome, anuran, amphibian

Abstract

Background

Cane toads (*Rhinella marina*) are an iconic invasive species introduced to four continents and well utilized for studies of rapid evolution in introduced environments. Despite the long introduction history of this species, its profound ecological impacts and its utility for demonstrating evolutionary principles, genetic information is sparse. Here we produce a *de novo* transcriptome spanning multiple tissues and life stages to enable investigation of the genetic basis of previously identified rapid phenotypic change over the introduced range.

Findings

Using approximately 1.9 billion reads, from developing tadpoles and 6 adult tissue-specific cDNA libraries, and a transcriptome assembly pipeline encompassing 100 separate *de novo* assemblies, we constructed 62,202 transcripts, of which we functionally annotated ~50 %. Our transcriptome assembly exhibits 90% full-length completeness of the BUSCO (benchmarking universal single-copy orthologs) dataset. Robust assembly metrics and comparisons to several available anuran transcriptomes and genomes indicate that our cane toad assembly is one of the most complete anuran genomic resources available.

Conclusion

This comprehensive anuran transcriptome will provide a valuable resource for investigation of genes under selection during invasion in cane toads, but will also greatly expand our general knowledge of anuran genomes, which are underrepresented in the literature. The dataset is publically available in NCBI and GigaDB to serve as a resource for other researchers.

Data Description

Background

It is well established that genome size across taxa is related to repetitive DNA content [1]. Highly repetitive genomes present significant challenges to genome assembly [2], which likely accounts for the scarcity of large genome sequences currently available. Anuran genome size is highly variable (C-values of 0.95-13.02; [3]) and, to date, genome sequences of only three anurans have been published: *Xenopus tropicalis* [4], *X. laevis* [5] and *Nanorana parkeri* [6]. Large genomes typify many Bufonids, including the cane toad (*Rhinella marina*; average reported C-value = 4.79 [3]) and none have been sequenced to date. Transcriptome sequencing provides a tenable alternative to genome sequencing in anurans because the large, repetitive, non-coding regions typical of their large genomes are not sequenced [7].

Cane toads are an excellent model for the study of invasion. Because they were intentionally and repeatedly introduced to novel environments as a biocontrol agent, their introduction history is well-documented [8]. A wealth of evolutionary and ecological knowledge about cane toads currently exists, documenting phenotypic evidence of rapid evolution in introduced environments, but genomic data are scarce [9]. Providing access to well-developed genomic resources for the cane toad will enable the investigation of the genetic basis of traits underlying invasion ability in this species, which will in turn significantly advance our understanding of invasion genetics for all species. Here we present a *de novo* transcriptome assembly covering multiple *R. marina* tissues and life stages, representing one of most complete anuran genomic resources reported to date.

Samples

Cane toad samples and tissues (seven in total) used in this study were obtained from several sources within the invasive (Australian) and native (Brazilian) range. Several different methods were used to prepare and sequence samples; for simplicity we describe the samples and data sources used based on the tissue types sequenced (Table 1). *Brain and Spleen* – four adult female toads were collected across two sites in Australia, two from Durack (15.9419° S, 127.2202° E), Western Australia, and two from Gordonvale (17.0972° S, 145.7792° E), Queensland, in May 2015. Toads were euthanized using lethal injection of 150mg/kg sodium pentobarbital and whole brain and spleen was harvested and immediately stored in RNAlater (Qiagen, USA), kept at 4 °C, then transferred to -80 °C for storage until RNA extraction. *Tadpoles* – We conducted a tadpole rearing experiment in March 2015. Four adult toads (2 males and 2 females) were collected from both Oombulgurri (15.1818° S, 127.8413° E), Western Australia, Australia and Innisfail (17.4963° S, 146.0465° E), Queensland, Australia. To obtain egg clutches, pairs of adult male and female toads per population (i.e. 2 separate male x female crosses) were subcutaneously injected with 0.25mg/ml Leuproelin acetate (Lucrin® Abbott Australasia, Kurnell, Australia) in amphibian Ringer's solution to stimulate spawning; males received 0.25 ml and females 0.75 ml. The pairs of male and female toads were left overnight in 750L plastic enclosures, containing bore water, to lay and fertilize egg clutches. Egg clutches were removed and placed in 17L tanks containing continuously aerated bore water and monitored to ensure fertilization had occurred. Embryos were selected once they reached Gosner stage 16-17 [10]. Three replicates of five fertilised embryos were removed per clutch and placed in 1L containers, each with 750ml bore water, where they were raised until 10 days old; water was changed daily and developing tadpoles were fed 12mg of a commercial algae supplement (Hikari algae pellets, Kyorin, Himeji City, Japan) after each water change. One tadpole from each of the three replicate tadpole tanks per clutch

was euthanized (immersion in 2g/L Tricaine methanesulfonate) and immediately stored in RNAlater, kept at 4 °C, then transferred to -80 °C for storage until RNA extraction.

Table 1. Cane toad samples used to generate the de novo reference transcriptome

Tissue	Origin	Platform	Sample ID (library size)	Sampling location	Sex	SRA
Brain	Australia	HiSeq 2500	B19 (23.9 M)	Durack	F	SRR5446736
		(2x125 bp)	B20 (27.7 M)	Durack	F	SRR5446735
			B31 (24.8 M)	Gordonvale	F	SRR5446734
			B32 (22.3 M)	Gordonvale	F	SRR5446733
Spleen	Australia	HiSeq 2500	S1 (23.8 M)	Gordonvale	F	SRR5446732
		(2x125 bp)	S2 (25.0 M)	Gordonvale	F	SRR5446732
			S18 (24.7 M)	Durack	F	SRR5446732
			S19 (23.6 M)	Durack	F	SRR5446732
Muscle	Australia	HiSeq 2000	RM0021M (93.8 M)	El Questro	F	SRR1910534
		(2x100 bp)				SRR1910535
				Purnululu	F	SRR1910543
			RM0094M (88.2 M)	Innisfail	F	SRR1910545
			RM0108M (97.6 M)	Rossville	F	SRR1910549
			RM0169M (80.0 M)			
Tadpole	Australia	HiSeq 2500	T1 (26.4 M)	Oombulgurri	Both	SRR5446728
		(2x125 bp)	T4 (24.5 M)	Oombulgurri	Both	SRR5446727
			T7 (23.2 M)	Innisfail	Both	SRR5446726
			T10 (25.7 M)	Innisfail	Both	SRR5446725
Liver	Brazil	HiSeq 2000 (2x75bp)	RMTP (536.8 M)	Macapá city	NA	SRR1514601
Ovary	Brazil	HiSeq 1500 (2x125 bp)	AR19 (434.1 M)	Macapá city	F	SRR5446724
Testes	Brazil	HiSeq 1500 (2x125 bp)	AR05 (410.5 M)	Macapá city	M	SRR5446723

Library size is given as raw sequenced reads in millions (M), sex donated as F - female, M – male, Both – sample contains mixed individuals of both sexes, NA – information unknown.

Total RNA was extracted from each of the brain, spleen and tadpole samples using Qiagen RNeasy kits (Qiagen, USA), following the manufacturers protocol with an additional DNase digestion step. Extracted RNA was quantified using a Quibit RNA HS assay on a Qubit 3.0 Fluorometer (Life technologies, USA). For the tadpole sequencing, total RNA from the three ‘replicate’ tadpoles per clutch were pooled in equal quantities, resulting in 4 pooled samples. Two µg of total RNA per sample were sent to Macrogen (Macrogen Inc., Seoul, ROK), where mRNA libraries were constructed using the TruSeq mRNA v2 sample kit (Illumina Inc, San Deigo, USA) that included a 300 bp size selection step. Libraries were sequenced on one lane of Illumina HiSeq 2500 (Illumina Inc, San Deigo, USA), generating 295.6 million paired-end 2 x 125bp reads. Raw reads are available in the NCBI Short Read Archive (SRA) under the Bioproject Accession: PRJNA382870.

Muscle – We downloaded raw fastq files (NCBI Bioproject Accession: PRJNA277985; paired-end 2 x 100bp; Illumina HiSeq-2000) for four adult female toads (RM0021M, RM0094M, RM0108M and RM0169M) across four populations in Australia (El Questro, 16.007872S, 128.020494E; Purnululu National Park, 17.4334° S, 128.3018° E, both Western Australia; Innisfail, 17.4963° S, 146.0465° E; Rossville, 15.7054° S, 145.2229° E, both Queensland) previously used to build a *de novo* muscle (*triceps femoris*) transcriptome [9].

Ovary and Testes – Two adult toads (1 male and 1 female) were collected from Macapá city (0.0432° S, 51.1241° W), Amapá state, Brazil in December 2015. Toads were euthanized as described above and ovary and testes were excised and immediately stored in RNAlater then kept at 4 °C for storage until RNA extraction. Total RNA was extracted using Qiagen RNeasy kits, following the manufacturers protocol with an additional DNase digestion step. Extracted RNA was quantified using a Qubit RNA BR assay and RNA integrity (RIN) was assessed

using a Tapestation 2200 (Aligent Tech., Santa Clara, CA, USA) with an RNA Screen. One μg of total RNA per sample was used to construct mRNA libraries using the TruSeq mRNA v2 sample kit, which included a 130–350 bp size selection step. Both libraries were run on a HiSeq 1500 using Illumina V4 PE chemistry across two lanes (one lane for each sample) generating 844.6 million paired-end 2 x 125bp reads. Raw reads are available in the NCBI SRA under the Bioproject Accession: PRJNA382870.

Liver – We downloaded raw fastq files (NCBI Bioproject Accession: PRJNA255079; paired-end 2 x 75 bp; Illumina HiSeq-2000) from a pool of five adult toads from Macapá city, Amapá state, Brazil, previously used to build a *de novo* liver transcriptome [11].

Data preprocessing and multiple *de novo* transcriptome assemblies

Raw reads from each sample were first processed with Trimmomatic v0.33 [12], using the following parameters: ILLUMINACLIP:TruSeq3-PE.fa:2:30:10:4 HEADCROP:13 AVGQUAL:30 MINLEN:36, to: *i*) remove adaptor sequences, *ii*) trim the first 13 bp of a read, *iii*) discard reads with an average quality < Phred 30 and *iv*) remove reads < 36bp after processing. We then concatenated reads into two input data sets, one containing all samples from Australia and one containing those from Brazil. To reduce the computational load of assembly we used the *in silico* normalization approach implemented in Trinity v2.1.1 [13], with --*normalize_max_read_cov=50*, on both of the input data sets. The normalized Australia and Brazil data sets contained ~42.2 million and ~82.2 million reads, respectively. Multiple independent *de novo* transcriptome assemblies were conducted for each of the input data sets, resulting in 100 separate assemblies (see Table 2). In brief, we used three assemblers: Trinity, with default parameters and --*min_contig_length=300*; SOAPdenovo-Trans v1.03 [14], with 13 different k-mers (apart from the Brazil input set which had 12) for each combination of

EdgeCovCutoff=2, *mergeLevel=1* and *EdgeCovCutoff=3*, *mergeLevel=2* - the parameters -f, -F and *minContigLen=200* were the same for all assemblies; velvet v1.2.09/oases v0.2.08 [15,16], with 12 different k-mers for each combination of -*cov_cutoff=3*, -*min_pair_count=4* and -*cov_cutoff=5*, -*min_pair_count=6*, where -*ins_length=300* and -*min_trans_lgth=200* were consistent across assemblies. The individual assemblies were then compiled into an 'over-assembly' of ~ 42 million transcripts. To reduce redundancy in the 'over-assembly' we used the tr2aacds pipeline from the Evidential Gene package [17], which selects the 'optimal' set of transcripts based on their coding potential. This reduced the redundant 'over-assembly' to the final assembly of 62,202 transcripts. Of these, 50 % (31,040) are commonly expressed among the seven different tissues used in the assembly, while a total of 6.64 % exhibit tissue specific expression (see Fig. S1: Additional file 1. and [32]). We then used TransDecoder v3.0.0 to predict protein coding sequences (CDS) with a minimum CDS of 100bp. Transvestigator [18] was used to prepare the final assembly for submission to NCBI's Transcriptome Shotgun Assembly (TSA) database – accessible through the PRJNA383966 accession. Results from the assembly pipeline are described in Table 3. As the 'dropset' – those transcripts not kept in the 'optimal' tr2aacds output - may contain other biologically relevant transcripts, such as non-coding RNAs and active transposable elements, we also provide these transcripts in the associated GigaDB repository [32~~1~~].

Table 2. *De novo* assembler parameters used to produce the 'over-assembly'

Assembler	*k*-mers	Parameter combinations	No. of assemblies
Trinity	25	Default	Aus 1, Brazil 1
SOAPdenovo-Trans	21, 25, 29, 33, 37, 41, 45, 49, 59, 69, 79, 89, 99 (No 99 for the Brazil input set)	*EdgeCovCutoff=2* and *mergeLevel=1;* *EdgeCovCutoff=3* and *mergeLevel=2*	Aus 13, Brazil 12; Aus 13, Brazil 12
Velvet/Oases	21, 25, 29, 33, 37, 41, 45, 49, 59, 69, 79, 89	*cov_cutoff=3* and *min_pair_count=4;*	Aus 12, Brazil 12; Aus 12, Brazil 12

	cov_cutoff=5 and *min_pair_count=6*	
		Total: 100

Annotation

We conducted functional annotation based on our predicted protein sequences utilizing the automated Trinotate pipeline. Transcripts were first annotated based on sequence homology, where assembled nucleotides and translated CDS sequences were used in BLASTx and BLASTp searches, against the UniProt/Swiss-Prot database (downloaded Feb. 2017) using a standalone version of blast v 2.2.26+ [19], with an e-value cutoff of 1x 10^{-5}. Pfam [20] functional domains (downloaded Feb. 2017) were identified in protein coding sequences using hmmscan [21]; signal peptides and transmembrane domains were assigned using hidden Markov model prediction implemented in SignalP v4.1 [22] and TMHMM v2.0c [23] respectively. Finally, transcripts were compared to curated annotations in eggNOG [24] and Gene Ontology (GO) [25] databases. A summary of annotation metrics is provided in Table 3. The combined Trinotate functional annotations to the TSA assembly are available in the associated GigaDB, see [32~~1~~].

Table 3. Summary of transcriptome assembly and annotation statistics compared to previous cane toad transcriptomes

	This study	Muscle[a]	Liver[b]
Assembly			
Filtered read pairs	945,348,780	99,462,214	265,684,605
in silico normalized reads	129,051,008	18,713,526	-
Assembly size (bp)	83,724,193	60,388,685	80,251,892
Number of transcripts	62,202	57,580	131,020
N50	2,377	1,871	916
Average length (bp)	1,346	1,049	613
Minimum length (bp)	297	201	201

Maximum length (bp)	99,438	40,546	17,369
Median length (bp)	698	577	331
GC %	46.05	45.06	44.32
Transcripts with CDS	62,202	19,751	-
Annotation			
Transcripts with BLASTx hit	31,103	21,533	-
Transcripts with BLASTp hit	28,560	16,754	-
Transcripts with GO terms	28,399	19,500	-

[a][9];[b][11]

Quality and completeness of the cane toad transcriptome

To evaluate our new multi-tissue transcriptome assembly, we used three comparative approaches to assess relative quality and completeness. First, we compared core assembly statistics of the new assembly to our two previous cane toad single-tissue transcriptomes derived from muscle and liver tissue (see Table 3). The inclusion of data from multiple tissues (encompassing a 9.5- and 3.5-fold increase in read input compared to the muscle and liver transcriptomes, respectively) resulted in increases of all assembly metrics, apart from the number of assembled transcripts, which fell compared to the liver transcriptome (Table 3). Notably, mean transcript length increased from 613 (liver) to 1,346 bp and transcript n50 increased from 916 (liver) to 2,377 bp. The total assembled bases were similar between the multi-tissue transcriptome and that assembled from liver, yet higher (~20 Million bp) than that produced from muscle tissue. ~~Importantly, the new multi-tissue assembly increases the coverage of transcripts containing protein coding sequences with associated Blast matches and Gene Ontology annotations.~~ Additionally, our assembly exhibits comparable lengths of mRNAs and CDS to those from *X. tropicalis* (gene build v9.0, downloaded Xenbase.org, Aug. 2017), see (Fig. S2: Additional file 1.), albeit with a greater frequency of shorter features. This is not unexpected given we are comparing a *de novo* transcriptome assembly to gene models from a genome assembly. Also, our assembly shows good coverage of the

Formatted: Font: Italic

lengths of CDS features compared to *X. tropicalis* (Fig. S3: Additional file 1.), given both species are substantially divergent. Importantly, the new *R. marina* multi-tissue assembly increases the coverage of transcripts containing protein coding sequences with associated Blast matches and Gene Ontology annotations compared to the previously available *R. marina* assemblies.

Second, we evaluated the new assembly using the BUSCO (benchmarking universal single-copy orthologs) vertebrate gene set [26] ~~(Table 4.)~~, which uses 3023 near-universal orthologs (hereafter BUSCOs) to evaluate relative completeness of assemblies and compared the results to the previous *R. marina* assemblies and those of several available amphibian transcriptomes and genomes (see Table 4.). We used BUSCO v1.2 [26] with the default e-value cutoff of 0.01 and *–mode=Trans* for all the transcriptomes compared and *–mode=OGS* for the genome comparisons (using the *N. parkeri* v2.0, *X. tropicalis* v9.0 and *X. laevis* v9.1_1.8.3.2 gene builds downloaded from Xenbase.org, Aug. 2017). ~~Compared to several available anuran transcriptomes and genomes~~ ~~o~~Our multi-tissue assembly had a much higher percentage of complete BUSCOs ~~genes~~ (90 %), apart from the two *Xenopus* ~~*tropicalis*~~ genomes which exhibited comparable results (*X. tropicalis,* 91~~2~~ % and *X. laevis, 97 %*). Additionally, our multi-tissue transcriptome has low ~~gene~~ BUSCO missingness, intermediate duplication of complete BUSCOs and the second lowest level of ~~of~~ fragmented BUSCOs~~ation~~. In contrast to the previous *R. marina* transcriptomes specifically, the new assembly has less fragmented and 20 – 30 % more complete BUSCO genes – suggesting the presence of more full-length transcripts. Overall the comparison of BUSCO results revealed our assembly to be one of the most complete references available for anurans.

Table 4. BUSCO analysis of transcriptome completeness

Formatted: Font: Italic
Formatted: Font: Italic
Formatted: Font: Italic
Formatted: Font: Italic
Formatted: Font: 12 pt
Formatted: Font: 12 pt
Formatted: Font: 12 pt
Formatted: Font: Italic
Formatted: Font: Italic

	Complete BUSCOs (%)	Complete and d~~D~~uplicated BUSCOs (%)	Fragmented BUSCOs (%)	Missing BUSCOs (%)
R. marina transcriptomes				
This study	90	4.7	1.7	7.8
Muscle[a]	60	4.6	5.7	33
Liver[b]	69	0.6	4.1	26
Select anuran transcriptomes				
Bufotes viridis[c]	26	0.3	15	57
Rana catesbeiana[d]	79	42	2.8	17
Pelohylax nigromaculatus[e]	50	0.4	7.8	41
Microhyla fissipes[f]	73	1.2	4.7	21
Select anuran genomes				
Xenopus laevis[g]	97~~61~~	51~~9~~	1.4~~2.9~~	1.4~~35~~
Xenopus tropicalis[h]	91~~2~~	4.1~~62~~	3.7~~1~~	4.9~~5~~
Nanorana parkeri[i]	76	2.8	9.0	14

[a][9],[b][11],[c][7],[d][27],[e][28],[f][29]~~,[g][4]~~,[g~~h~~][5],[h][4], [i][6] Complete BUSCOs refer to those with a full-length match in the assembly; Complete and duplicated refers to those BUSCOs that are complete within an assembly but have multiple matches present; Fragmented are those BUSCOs that only have a partial match in the assembly and Missing refers to those BUSCOs that do not have a corresponding match in the assembly.

Third, we compared the multi-tissue transcriptome to the previous cane toad transcriptomes and the ~~*X. tropicalis* protein se~~three currently available Anuran genomes ~~t~~ through both standard and reciprocal best-hit BLAST approaches. The standard approach revealed 40,741 (65.5 %) and 31,189 (50.1 %) of our new assembly had significant matches (e-value <10^{-3}) to the liver and muscle transcriptomes, respectively. The reciprocal best-hit approach reduced the number of significant matches to both the liver (23,943; 38.5 %) and muscle (15,892; 25.5%) transcriptomes, which may in part be due to transcripts mapping to multiple isoforms in the different assemblies. This, together with the high number of protein-coding transcripts in the multi-tissue assembly, indicates the new assembly still contains some redundancy and

Formatted Table

that we have assembled multiple transcripts variants for some genes. Standard BLAST comparisons~~s~~ of our assembly to the ~~the~~ *X. tropicalis*, *X. laevis* and *N. parkeri* p~~p~~roteins exhibited ~~38,878~~40,275 (64~~2~~.7~~5~~ %), 40,218 (64.7 %) and 40,244 (64.7 %) significant matches, respectively; 37,064 of our assembled transcripts with significant matches are common to all three species (Fig. S4: Additional file 1.).~~,~~ ~~while the reciprocal best-hit approach yielded 13,941 (22.4 %) putative orthologs between the two species~~Of our 31,103 assembled transcripts with annotations, 97.8 % (30,423) have significant matches to *X. tropicalis*, while 97.9 % (30,465) have matches to *X. laevis* and 98.3 % (30,574) to *N. parkeri*; 96.3 % (29,967) are common to all three species (Fig. S5: Additional file 1.). The high percentage of annotated transcripts with matches to *X. tropicalis*, *X. laevis* and *N. parkeri* provides further evidence that our assembly pipeline produced transcripts with strong anuran homology.

Identification of Anuran Orthologues

Current efforts to identify amphibian-specific genes has been hampered by a lack of high quality full-length genes for numerous amphibian species [30]. So far, the identification of amphibian-specific genes has not been possible as orthologous counterparts have only been identified between the two *Xenopus* genomes. We used OrthoFinder v1.1.10 [31] with default parameters to identify orthologues between our newly assembled *R. marina* CDS containing transcripts and the proteomes from *N. parkeri*, *X. tropicalis* and *X. laevis* (using the same gene build versions as in the BUSCO analysis). OrthoFinder identifies ‘orthogroups’ (a group of genes descended from a single gene in the last common ancestor of a group of species [31]) and then orthologues between each pair of species in the comparison. Because OrthoFinder classifies genes with multiple orthologues (i.e. many to many relationships) in ‘orthogroups’

Formatted: Heading 2

Formatted: Font: Italic

it may reduce the impact multiple isoforms of the same gene have in such analyses. We assigned 60.2 % (94,516) of all the genes examined to 18,776 ‘orthogroups’ (see Additional file 2.), of which 50% of all genes were found in ‘orthogroups’ with four or more genes. Additionally, we identified 12,674 ‘orthogroups’ which contained genes from all four species and 4,586 of these consisted entirely of single-copy genes. The dataset presented here may be useful for further research into identifying amphibian-specific genesaid this effort, so we have included these orthologs is analysis in its entirety in the associated GigaDB repository [321].

Conclusions

This comprehensive anuran transcriptome will not only serve as a valuable reference for investigation of genes under selection during invasion in cane toads, but will also expand our general knowledge of anuran genomes. Additionally, we have identified numerous orthologous transcripts to *X. tropicalis*, *X. laevis* and *N. parkeri* proteins, which may aid the identification of amphibian-specific genes – an important objective of AmphiBase [30].

Abbreviations

SRA: Short Read Archive; CDS: coding sequence; TSA: Transcriptome Shotgun Assembly; GO: Gene Ontology; BUSCO: Benchmarking universal single copy orthologs

Availability of supporting data

The datasets supporting the results presented here are available in the associated GigaDB repository [31]. All raw sequencing data used in this study is available in the SRA and associated with the following BioProject accessions: PRJNA277985, PRJNA255079, PRJNA382870 and PRJNA383966. The final transcriptome assembly has been deposited at

DDBJ/EMBL/GenBank under the accession GFMT00000000. The version described in this paper is the first version, GFMT01000000.

Declarations

Acknowledgements

We thank Serena Lam and Chris Jolly for assistance with sample collection. This project was funded through: An Australian Research Council (ARC) Discovery Early Career Research Award (DE150101393) to LAR, ARC Laureate Fellowship to RS (FL120100074), Centre for Integrative Ecology research funds, Deakin University to MFR, FCT Investigator grant to MC [IF/00283/2014/CP1256/CT0012], Research project and research fellowship (PQ 10/2012 and Universal 14-2013) and post-doctoral fellowship to MV (232916/2013-6, CNPq) and FEDER (COMPETE, POCI-01-0145-FEDER-006821) and FCT (UID/BIA/50027/2013) funds to FS.

Competing interests

We declare no competing interests.

Ethics statement

Ethics approval for the capture of wild Australian samples was provided under the University of Sydney permit 2014/562, the rearing of tadpoles by the University of Sydney permit 2013/6033 and Brazilian samples under the Brazilian Federal Chico Mendes Institute for Biodiversity Conservation (ICMBio), through license number 38047-3.

Author contributions

MFR, MV and LAR collected animals and conducted the sample preparation. JGR and MRC conducted tadpole rearing. MFR, FS, DMS, MC and LAR conducted RNA isolation for sequencing and library construction. AJW contributed samples. MFR conducted transcriptome assemblies and analysis. MFR, FS, RS and LAR wrote the manuscript and participated in study design. All authors commented on the manuscript and approved the final submission.

References

1. Kidwell MG. Transposable elements and the evolution of genome size in eukaryotes. Genetica. 2002;115:49–63.

2. Treangen TJ, Salzberg SL. Repetitive DNA and next-generation sequencing: computational challenges and solutions. Nat. Rev. Genet. 2011;13:36–46.

3. Gregory T. Animal Genome Size Database. 2015 http://www.genomesize.com Accessed 3 March 2017.

4. Hellsten U, Harland RM, Gilchrist MJ, Hendrix D, Jurka J, Kapitonov V, et al. The Genome of the Western Clawed Frog Xenopus tropicalis. Science. 2010;328:633–6.

5. Session AM, Uno Y, Kwon T, Chapman JA, Toyoda A, Takahashi S, et al. Genome evolution in the allotetraploid frog Xenopus laevis. Nature. 2016;538:336–43.

6. Sun Y-B, Xiong Z-J, Xiang X-Y, Liu S-P, Zhou W-W, Tu X-L, et al. Whole-genome sequence of the Tibetan frog Nanorana parkeri and the comparative evolution of tetrapod genomes. Proc. Natl. Acad. Sci. U. S. A. 2015;112:E1257-62.

7. Gerchen JF, Reichert SJ, Röhr JT, Dieterich C, Kloas W, Stöck M. A Single Transcriptome of a Green Toad (Bufo viridis) Yields Candidate Genes for Sex Determination and -Differentiation and Non-Anonymous Population Genetic Markers. PLoS One. 2016;11:e0156419.

8. Kraus F. Alien reptiles and amphibians : a scientific compendium and analysis. Springer; 2009.

9. Rollins LA, Richardson MF, Shine R. A genetic perspective on rapid evolution in cane

toads (*Rhinella marina*). Mol. Ecol. 2015;24:2264–76.

10. Gosner KL. A Simplified Table for Staging Anuran Embryos and Larvae with Notes on Identification. Herpetologica. 1960;16:183–90.

11. Arthofer W, Banbury BL, Carneiro M, Cicconardi F, Duda TF, Harris RB, et al. Genomic Resources Notes Accepted 1 August 2014-30 September 2014. Mol. Ecol. Resour. 2015;15:228–9.

12. Bolger AM, Lohse M, Usadel B. Trimmomatic: a flexible trimmer for Illumina sequence data. Bioinformatics. 2014;30:2114–20.

13. Grabherr MG, Haas BJ, Yassour M, Levin JZ, Thompson DA, Amit I, et al. Full-length transcriptome assembly from RNA-Seq data without a reference genome. Nat. Biotechnol. 2011;29:644–52.

14. Xie Y, Wu G, Tang J, Luo R, Patterson J, Liu S, et al. SOAPdenovo-Trans: de novo transcriptome assembly with short RNA-Seq reads. Bioinformatics. 2014;30:1660–6.

15. Zerbino DR, Birney E. Velvet: algorithms for de novo short read assembly using de Bruijn graphs. Genome Res. 2008;18:821–9.

16. Schulz MH, Zerbino DR, Vingron M, Birney E. Oases: robust de novo RNA-seq assembly across the dynamic range of expression levels. Bioinformatics. 2012;28:1086–92.

17. Gilbert D. Accurate & complete gene construction with EvidentialGene. F1000Research. 2016;5.

18. DeRego T, Hall B, Ben-guin, Geib S. Transvestigator early release. Zenodo. 2014.

19. Camacho C, Coulouris G, Avagyan V, Ma N, Papadopoulos J, Bealer K, et al. BLAST+: architecture and applications. BMC Bioinformatics. 2009;10:421.

20. Finn RD, Bateman A, Clements J, Coggill P, Eberhardt RY, Eddy SR, et al. Pfam: the protein families database. Nucleic Acids Res. 2014;42:D222-30.

21. Eddy SR, Crooks G, Green R, Brenner S, Altschul S. Accelerated Profile HMM Searches. Pearson WR, editor. PLoS Comput. Biol. 2011;7:e1002195.

22. Petersen TN, Brunak S, von Heijne G, Nielsen H. SignalP 4.0: discriminating signal peptides from transmembrane regions. Nat. Methods. 2011;8:785–6.

23. Krogh A, Larsson B, von Heijne G, Sonnhammer EL. Predicting transmembrane protein topology with a hidden Markov model: application to complete genomes. J. Mol. Biol. 2001;305:567–80.

24. Powell S, Szklarczyk D, Trachana K, Roth A, Kuhn M, Muller J, et al. eggNOG v3.0: orthologous groups covering 1133 organisms at 41 different taxonomic ranges. Nucleic Acids Res. 2012;40:D284–9.

25. Ashburner M, Ball CA, Blake JA, Botstein D, Butler H, Cherry JM, et al. Gene Ontology: tool for the unification of biology. Nat. Genet. 2000;25:25–9.

26. Simão FA, Waterhouse RM, Ioannidis P, Kriventseva E V., Zdobnov EM. BUSCO: assessing genome assembly and annotation completeness with single-copy orthologs. Bioinformatics 2015;31:3210–2.

27. Birol I, Behsaz B, Hammond SA, Kucuk E, Veldhoen N, Helbing CC. De novo Transcriptome Assemblies of Rana (Lithobates) catesbeiana and Xenopus laevis Tadpole Livers for Comparative Genomics without Reference Genomes. Plateroti M, editor. PLoS One 2015;10:e0130720.

28. Huang L, Li J, Anboukaria H, Luo Z, Zhao M, Wu H. Comparative transcriptome analyses of seven anurans reveal functions and adaptations of amphibian skin. Sci. Rep. 2016;6:24069.

29. Zhao L, Liu L, Wang S, Wang H, Jiang J. Transcriptome profiles of metamorphosis in the ornamented pygmy frog Microhyla fissipes clarify the functions of thyroid hormone receptors in metamorphosis. Sci. Rep. 2016;6:27310.

30. Kwon T. AmphiBase: A new genomic resource for non-model amphibian species. genesis. 2017;55:e23010.

31. Emms DM, Kelly S. OrthoFinder: solving fundamental biases in whole genome comparisons dramatically improves orthogroup inference accuracy. Genome Biology 2015;16:157

321. Richardson MF, Sequeira F, Sclechnik D, Carneiro M, Vallinoto M, Reid JG, et al. Supporting data for "Improving amphibian genomic resources: a multi-tissue reference transcriptome of an iconic invader" GigaDB doi XXX

Formatted: Font: Times New Roman

Formatted: Line spacing: 1.5 lines

Formatted: Font: Times New Roman, Not Italic

Formatted: Font: Times New Roman

Formatted: Font: Times New Roman, Not Bold

Formatted: Font: Times New Roman

Additional file 1

Click here to access/download
Supplementary Material
Supplemental_Figures.docx

Additional file 2

Click here to access/download
Supplementary Material
TableS1.xlsx

Data info

Click here to access/download
Supplementary Material
Richardson_etal_gigascience_datainfo.docx

Click here to access/download
Supplementary Material
README.txt
