# Supplementary material for: Improving amphibian genomic resources: a multitissue reference transcriptome of an iconic invader
Source: Gigascience. 2017 Nov 27;7(1):gix114. doi: 10.1093/gigascience/gix114 (PMC5765561; doi:10.1093/gigascience/gix114)
Supplement: Supplement Figures [file gix114_supp.docx]

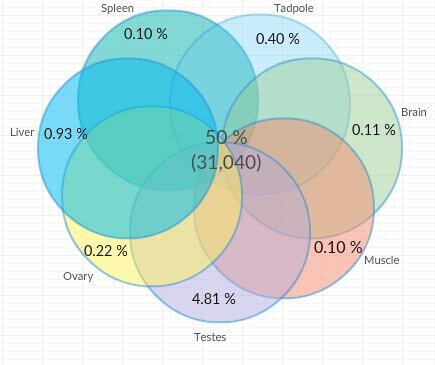


**Figure S1.** Schematic diagram showing the percentage (%) of expressed transcripts among the 7 different tissues used in the assembly. For brevity, we only show those common to all tissues (centre of elements) and those uniquely expressed in each separate tissue. Transcript expression was quantified using Salmon v0.8.0 [1] using *–l IU* and default parameters. Additionally, detailed comparative analysis of expression among all tissue combinations is provided in the associated GigaDB repository [2].

**Figure S2.** Histogram of the lengths of *R. marina* assembled mRNAs and CDS compared to those from *X. tropicalis* (gene build v9.0, Xenbase.org, Aug. 2017)

**Figure S3.** Scatterplot showing the coverage of each *R. marina* CDS length in base pairs (bp) compared to the corresponding CDS match in *X. tropicalis* (gene build v9.0, Xenbase.org, Aug. 2017). CDS length matches were extracted from blastx queries with *evalue=10E-3* and *–max_target_seqs=1*


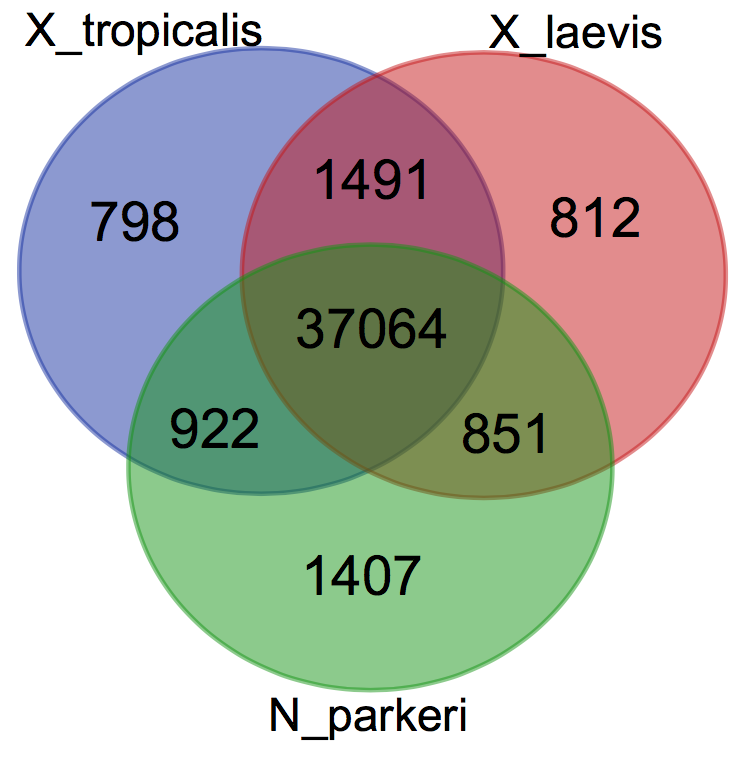


**Figure S4.** Venn diagram showing an overview of the significant Blastx matches (e-value <10^-3^) for our *R. marina* assembly against the proteins from *X. tropicalis, X. laevis* and *N. parkeri*. Venn diagram build using the Venn diagram webserver: http://bioinformatics.psb.ugent.be/webtools/Venn/


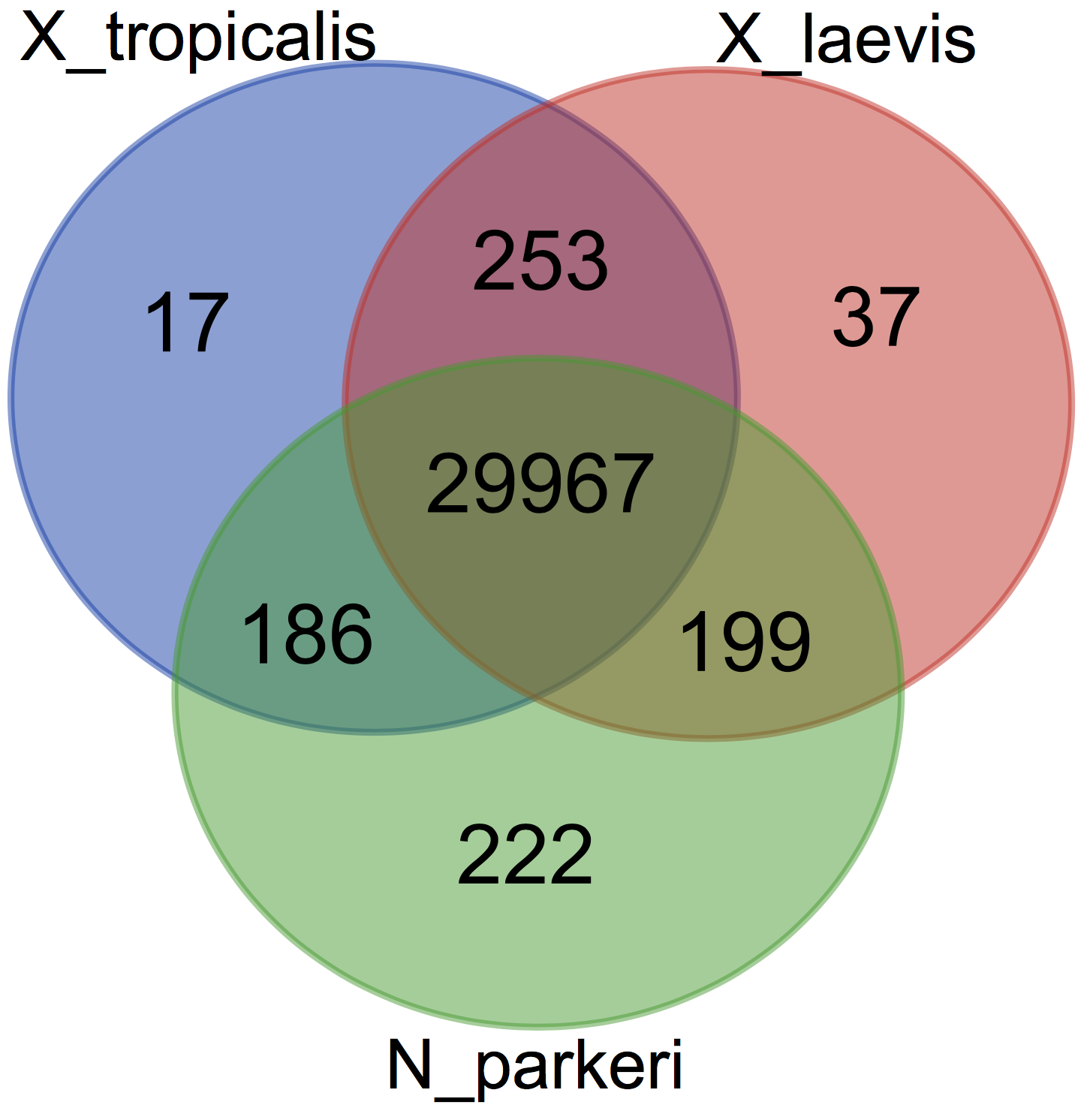


**Figure S5.** Venn diagram showing an overview of the significant Blastx matches (e-value <10^-3^) for our *R. marina* transcripts with annotations (31,103) against proteins from *X. tropicalis, X. laevis* and *N. parkeri*. Venn diagram build using the Venn diagram webserver: http://bioinformatics.psb.ugent.be/webtools/Venn/

**References:**

1. Patro R, Duggal G, Love MI, Irizarry RA, Kingsford C. Salmon provides fast and bias-aware quantification of transcript expression. Nature Methods. 2017; 14:417–419

2. Richardson MF, Sequeira F, Sclechnik D, Carneiro M, Vallinoto M, Reid JG, et al. Supporting data for "Improving amphibian genomic resources: a multi-tissue reference transcriptome of an iconic invader" GigaDB doi XXX
