# Supplementary material for: Improving amphibian genomic resources: a multitissue reference transcriptome of an iconic invader
Source: Gigascience. 2017 Nov 27;7(1):gix114. doi: 10.1093/gigascience/gix114 (PMC5765561; doi:10.1093/gigascience/gix114)
Supplement: Response-to-Reviewer-Comments_Original-Submission.pdf [file gix114_response-to-reviewer-comments_original-submission.pdf]

Author's Response To Reviewer Comments

Dear Dr Edmunds,

Thank you for the opportunity to submit a revised manuscript. We thank the reviewers for their positive and helpful comments that were valuable in revising and improving the manuscript. We have responded to all the reviewers' comments and incorporated changes into the revised manuscript.

Please find below our specific responses to the reviewer's comments and details of the changes carried out to the manuscript. We have also made additional effort within both the manuscript and associated data to conform to MINSEQE guidelines. As such, we have additional datasets we would like to include in GigaDB. Our lab protocols follow standard manufacturer protocols but we are happy to reproduce these in protocols.io if required.

We have uploaded a 'marked' manuscript will all changes shown in track changes (MS Word) and a clean version of the manuscript.

We confirm that this manuscript has not been published elsewhere and is not under consideration by another journal. All authors approved the manuscript and agree with its submission. We hope that our revised manuscript will meet the criteria needed for publication in Gigascience and we look forward to hearing from you.

Detailed response to reviewers:

NOTE: square brackets donate the line numbers corresponding to changes made in the track changes document.

Reviewer #1:

Richardson et al., reported the comprehensive transcriptome of cane toad Rhinella marina derived from the RNA-seq data of seven different tissues (some of them are from previously published data). Due to the estimated large genome size and limited availability of amphibian genomic resources, as the authors pointed out, I agree that this result would be useful resources in the community. However, there are some information missing to interpret their result.

(1) Although the authors described the detailed information about the samples, I cannot find the corresponding sample IDs in Table 1. Please provide the sampling location information and gender information in Table 1.

Response: This information has now been added to Table 1.

(2) Table 4 pointed out that this study provided 20-30% more transcriptome, compared to previously reported data (muscle & liver; 90% vs 60-69%). I wonder why. Is it because of brain samples, which may contain many neuro-specific genes? Or is it just general improvement of the data and/or analysis method? By discussing the function of "newly identified" genes in this

study, I think the authors can infer the reason.

Response: We believe it is due to general improvement, 1) by the addition of more data, and 2) a more robust and thorough assembly methodology. If it was due to the addition of a single tissue (such as brain) we would expect to see many tissue-specific genes expressed. Figure S1;additional file 1 (as requested by reviewer 3) shows that this is not the case, with the largest set of tissue specific expression only accounting for ~5% (testes) of transcript expression (total tissue specific expression is < 7 %).

(3) How many X. tropicalis (or human) orthologs are identified? Reciprocal best hits may not be good way to compare, because it is hard to reduce the isoforms in transcriptome data without a genome.

Response: We agree there are issues with reciprocal best hits as a method for identifying orthologues. As such, we have included a more thorough method (OrthoFinder) for identifying orthologues between the three available anuran genomes and our assembly. While there is still an issue with isoforms in the transcriptome data, the use of 'orthogroups' in this program allows for multiple isoforms to be included within an orthologous group – something not possible with the reciprocal best hit method. Using this method, we have identified 13,657 orthologues between R. marina and X. tropicalis and 4,586 that are found as single copy orthologues between all four species. More specifics are provided in the manuscript and we have made this analysis available in the associated public data repository. [293-313]

(4) It is not clear how the authors run BUSCO for comparison. For example, which version of X. laevis & X. tropicalis sequences are used?

Response: We have provided clarification of how this analysis was run [221-227] and given the versions of all gene builds used in the legend of Table 4 and the main text [226] .

Reviewer #2:

The study provides an expanded transcriptome resource for the marine toad, expanding genomic resources available for amphibians, which in general are underrepresented among genomic datasets. The study generated RNA-Seq datasets for brain, spleen, tadpoles, ovary, and testis in the marine toad and makes use of existing datasets for liver and muscle. The detailed methods documenting how samples were collected, and origin of samples were fantastic to read and should be a standard for others.

The authors use several assemblers to analyze the data, employing a variety of kmer sizes for some tools but not for Trinity. This is understandable for the version of Trinity used in the manuscript, however newer versions of Trinity permit changing kmer sizes. A newer version of Trinity might be useful for their analyses and help in development of their over-assembly. Arguably however, their use of multiple assemblers is likely very robust and was nice to read.

Response: Thank you for highlighting that new versions of Trinity now permit the changing of k-mers. As the reviewer states, because of the use of multiple assemblers (including those with the

option to vary k-mers) and multiple parameter sets we feel that further inclusion of more assemblies into the over-assembly (which already contains 100 separate assemblies) is unlikely to provide a more robust final assembly.

In general, I found the manuscript to be appropriate as a data note as there is no question these data will be useful. The comparison to other anuran transcriptomes is somewhat limited given the many more amphibian transcriptomes datasets that currently exist. More tissues would be useful beyond the addition of brain, spleen, tadpole and gonad which does not expand the resource much and but should be a useful advance for others.

Response: While more separate tissues would be nice to include in the assembly it is currently beyond our means for this project. We have included whole tadpoles in the assembly, which does contain all tissues (albeit it during a developmental stage). We have also included a more detailed comparison to other anuran resources (please see specific responses to reviewer 1 and 3) and included a more thorough investigation of anuran orthologues [293-313].

Reviewer #3:

The read number of each sample is sufficient (more than 1 M), and the number of genes is reasonable, but the quality control of de novo assembly is insufficient.

Major comments:
The authors need more quality controls as follows:
i) Histograms of lengths of mRNAs and CDSs compared with X. tropicalis.

Response: This is now provided as Figure S2 (additional file 1) and discussed in the main text [205-209].

ii) Coverage of the length of each CDS compared with that of X. tropicalis, using a scatter plot, for example.

Response: This is now provided as Figure S3 (additional file 1) and discussed in the main text [209-211].

iii) Comparison of all annotated transcripts with those of X. tropicalis, X. laevis, and Nanorana

Response: This is now provided as Figure S5 (additional file 1) and discussed in the main text [286-291].

iv) Blastx analysis of all transcripts for those of X. tropicalis and X. laevis.

Response: This is now provided as Figure S4 (additional file 1) and discussed in the main text [264-286].

v) Comparison of all transcripts between the 6 adult tissues and tadpoles of Rhinella marina

(cane toad) to show the percentages of common and tissue-specific genes in each tissue using a Venn diagram.

Response: We have been unable to construct a meaningful Venn/Euler diagram which shows all of the combinations between our 7 tissues that would be interpretable. We agree with the reviewer that this is an important comparison to include, so we have produced a simpler schematic showing the percentage of genes common to all tissues and the percentage unique to each tissue (as per the reviewer's main request). Additionally, we have included a text file which contains the full set of comparisons in the associated data repository. We present this data in brief in the main text and included the figure in the supplement as Figure S1(additional file 1) [161-163].

Minor comments

Table 4: Either Table 4 or its legend needs to be revised, because laevis (g) is Session et al. (5); and tropicalis (h) is Hellsten et al. (4). Which one is correct, the table, legend, or reference?

Response: Corrected – the legend citation was incorrect and has been revised.

If X. laevis is not mislabeled, the "Complete" (61%) seems to be too low compared to X. tropicalis (92%). Because X. laevis is allotetraploid, how did the authors calculate the completeness? Did the authors use the latest version of X. laevis gene models?

Response: We have updated this analysis to include the latest (as of 30th August 2017 - http://www.xenbase.org/other/static/ftpDatafiles.jsp) gene models for both Xenopus species and they both now show comparable results [Table 4, 237]. Completeness is calculated using the BUSCO pipeline and we have made this more explicit in the text – "Complete" refers to those BUSCOs that have a full-length match within the assembly. [228, 249-253]

The "Duplicated" is 59% (X. laevis) or 62% (X. tropicalis), which are much higher than the others. How is it so? The definition of these two terms (Complete and Duplicated) and others must be clarified and explained in the manuscript.

Response: We point the reviewers to the new results of this analysis, which show lower levels of complete BUSCOs that are duplicated. We also clarify these terms in the manuscript and provide standalone definitions in the Table legend for clarity. [230-232, Table 4]
