# Supplementary material for: Improving amphibian genomic resources: a multitissue reference transcriptome of an iconic invader
Source: Gigascience. 2017 Nov 27;7(1):gix114. doi: 10.1093/gigascience/gix114 (PMC5765561; doi:10.1093/gigascience/gix114)
Supplement: Response-to-Reviewer-Comments_Revision-1.pdf [file gix114_response-to-reviewer-comments_revision-1.pdf]

Author's Response To Reviewer Comments

Dear Scott,

Thank you, we are excited to have our paper published in GigaScience. Please find our comments to the requests below - we have made all the required changes.

Cheers,
Mark

I have already added the DOI and some RRID identifiers into the draft (I will send this in a follow up email) so please check you are happy with the changes. On top of these can you also carry out the following:

Response: We are happy with the changes.

1. Check the DOI mockup and if you are happy with it we will mint it and switch live:

Response: We are happy with the page.

2. Include ORCID identifiers for the corresponding author and any other authors that have them (the corresponding authors can login and verify them on the submission system, but for the other authors you can add them to the title page alongside affiliations).

Response: Added to title page

3. Include an image of the species in question as a figure.

Response: Now included as figure 1.

4. Double check for a final time you meet all our formatting requirements and reporting standards such as MINSEQE for the transcriptomics and ARRIVE for animal research.

Response: We have done as requesting and think we meet all of the requirements.

Reviewer reports:
'
Reviewer #1: The authors addressed most of my comments in this revised manuscript, so I am pleased to recommend it for GigaScience.

Response: Thank you.

Reviewer #2: The authors sufficiently addressed all comments in the revision.

Response: Thank you.
