# Supplementary material for: Improving amphibian genomic resources: a multitissue reference transcriptome of an iconic invader
Source: Gigascience. 2017 Nov 27;7(1):gix114. doi: 10.1093/gigascience/gix114 (PMC5765561; doi:10.1093/gigascience/gix114)
Supplement: Reviewer-1-Report-(Original-Submission) -- Taejoon Kwon — 04 Jun 2017 Reviewed [file gix114_reviewer-1-report-(original-submission).pdf]

**Reviewer Report**

**Title:** Improving amphibian genomic resources: a multi-tissue reference transcriptome of an iconic invader

**Version:** Original Submission **Date:** 6/4/2017

**Reviewer name:** Taejoon Kwon

**Reviewer Comments to Author:**

Richardson et al., reported the comprehensive transcriptome of cane toad Rhinella marina derived from the RNA-seq data of seven different tissues (some of them are from previously published data). Due to the estimated large genome size and limited availability of amphibian genomic resources, as the authors pointed out, I agree that this result would be useful resources in the community. However, there are some information missing to interpret their result.(1) Although the authors described the detailed information about the samples, I cannot find the corresponding sample IDs in Table 1. Please provide the sampling location information and gender information in Table 1.(2) Table 4 pointed out that this study provided 20-30% more transcriptome, compared to previously reported data (muscle & liver; 90% vs 60-69%). I wonder why. Is it because of brain samples, which may contain many neuro-specific genes? Or is it just general improvement of the data and/or analysis method? By discussing the function of "newly identified" genes in this study, I think the authors can infer the reason.(3) How many X. tropicalis (or human) orthologs are identified? Reciprocal best hits may not be good way to compare, because it is hard to reduce the isoforms in transcriptome data without a genome.(4) It is not clear how the authors run BUSCO for comparison. For example, which version of X. laevis & X. tropicalis sequences are used?

**Level of Interest**

Please indicate how interesting you found the manuscript: An article of importance in its field

**Quality of Written English**

Please indicate the quality of language in the manuscript: Acceptable

**Declaration of Competing Interests**

Please complete a declaration of competing interests, considering the following questions:

- Have you in the past five years received reimbursements, fees, funding, or salary from an organisation that may in any way gain or lose financially from the publication of this manuscript, either now or in the future?
- Do you hold any stocks or shares in an organisation that may in any way gain or lose financially from the publication of this manuscript, either now or in the future?

- Do you hold or are you currently applying for any patents relating to the content of the manuscript?
- Have you received reimbursements, fees, funding, or salary from an organization that holds or has applied for patents relating to the content of the manuscript?
- Do you have any other financial competing interests?
- Do you have any non-financial competing interests in relation to this paper?

If you can answer no to all of the above, write 'I declare that I have no competing interests' below. If your reply is yes to any, please give details below.

I declare that I have no competing interests.

I agree to the open peer review policy of the journal. I understand that my name will be included on my report to the authors and, if the manuscript is accepted for publication, my named report including any attachments I upload will be posted on the website along with the authors' responses. I agree for my report to be made available under an Open Access Creative Commons CC-BY license (http://creativecommons.org/licenses/by/4.0/). I understand that any comments which I do not wish to be included in my named report can be included as confidential comments to the editors, which will not be published.

I agree to the open peer review policy of the journal
