# Supplementary material for: Improving amphibian genomic resources: a multitissue reference transcriptome of an iconic invader
Source: Gigascience. 2017 Nov 27;7(1):gix114. doi: 10.1093/gigascience/gix114 (PMC5765561; doi:10.1093/gigascience/gix114)
Supplement: Reviewer-3-Report-(Original-Submission) -- Masanori Taira — 14 Jun 2017 Reviewed [file gix114_reviewer-3-report-(original-submission).pdf]

Reviewer Report

Title: Improving amphibian genomic resources: a multi-tissue reference transcriptome of an iconic invader

Version: Original Submission **Date:** 6/14/2017

Reviewer name: Masanori Taira

Reviewer Comments to Author:

The read number of each sample is sufficient (more than 1 M), and the number of genes is reasonable, but the quality control of de novo assembly is insufficient.

Major comments

The authors need more quality controls as follows:
i) Histograms of lengths of mRNAs and CDSs compared with X. tropicalis.
ii) Coverage of the length of each CDS compared with that of X. tropicalis, using a scatter plot, for example.
iii) Comparison of all annotated transcripts with those of X. tropicalis, X. laevis, and Nanorana
iv) Blastx analysis of all transcripts for those of X. tropicalis and X. laevis.
v) Comparison of all transcripts between the 6 adult tissues and tadpoles of Rhinella marina (cane toad) to show the percentages of common and tissue-specific genes in each tissue using a Venn diagram.

Minor comments
Table 4: Either Table 4 or its legend needs to be revised, because laevis (g) is Session et al. (5); and tropicalis (h) is Hellsten et al. (4). Which one is correct, the table, legend, or reference?
If X. laevis is not mislabeled, the "Complete" (61%) seems to be too low compared to X. tropicalis (92%). Because X. laevis is allotetraploid, how did the authors calculate the completeness? Did the authors use the latest version of X. laevis gene models?
The "Duplicated" is 59% (X. laevis) or 62% (X. tropicalis), which are much higher than the others. How is it so? The definition of these two terms (Complete and Duplicated) and others must be clarified and explained in the manuscript.

Level of Interest

Please indicate how interesting you found the manuscript: An article of importance in its field

Quality of Written English

Please indicate the quality of language in the manuscript: Acceptable

Declaration of Competing Interests

Please complete a declaration of competing interests, considering the following questions:

- Have you in the past five years received reimbursements, fees, funding, or salary from an organisation that may in any way gain or lose financially from the publication of this manuscript, either now or in the future?
- Do you hold any stocks or shares in an organisation that may in any way gain or lose financially from the publication of this manuscript, either now or in the future?
- Do you hold or are you currently applying for any patents relating to the content of the manuscript?
- Have you received reimbursements, fees, funding, or salary from an organization that holds or has applied for patents relating to the content of the manuscript?
- Do you have any other financial competing interests?
- Do you have any non-financial competing interests in relation to this paper?

If you can answer no to all of the above, write 'I declare that I have no competing interests' below. If your reply is yes to any, please give details below.

I declare that I have no competing interests.

I agree to the open peer review policy of the journal. I understand that my name will be included on my report to the authors and, if the manuscript is accepted for publication, my named report including any attachments I upload will be posted on the website along with the authors' responses. I agree for my report to be made available under an Open Access Creative Commons CC-BY license (http://creativecommons.org/licenses/by/4.0/). I understand that any comments which I do not wish to be included in my named report can be included as confidential comments to the editors, which will not be published.

I agree to the open peer review policy of the journal
